# Differences in the mechanical unfolding pathways of apo- and copper-bound azurins

**DOI:** 10.1038/s41598-018-19755-7

**Published:** 2018-01-31

**Authors:** Anju Yadav, Sanjoy Paul, Ravindra Venkatramani, Sri Rama Koti Ainavarapu

**Affiliations:** Department of Chemical Sciences, Tata Institute of Fundamental Research, Dr. Homi Bhabha Road, Colaba Mumbai, 400005 India

## Abstract

Metalloproteins carry out diverse biological functions including metal transport, electron transfer, and catalysis. At present, the influence of metal cofactors on metalloprotein stability is not well understood. Here, we report the mechanical stability and unfolding pathway of azurin, a cupredoxin family protein with β-barrel topology and type I copper-binding centre. Single-molecule force spectroscopy (SMFS) experiments reveal 2-state and 3-state unfolding pathways for apo-azurin. The intermediate in the 3-state pathway occurs at an unfolding contour length of 7.5 nm from the native state. Steered molecular dynamics (SMD) simulations show that apo-azurin unfolds *via* a first transition state (TS) where β2Β–β8 and β7–β8 strand pairs rupture to form the intermediate, which subsequently unfolds by the collective rupture of remaining strands. SMFS experiments on holo-azurin exhibit an additional 4-state pathway besides the 2-state and 3-state pathways. The unfolding contour length leading to the first intermediate is 6.7 nm suggesting a sequestration of ~1 nm polypeptide chain length by the copper. SMD simulations reveal atomistic details of the copper sequestration and predict a combined β4–β7 pair and copper coordination sphere rupture to create the third TS in the 4-state pathway. Our systematic studies provide detailed mechanistic insights on modulation of protein mechanical properties by metal-cofactors.

## Introduction

In general, protein folding and unfolding are complex processes governed by the underlying energy landscape and may exhibit parallel pathways, multiple intermediates and transition states (TSs)^1–3^. Further, the diversity of cooperative effects which create delocalized or collective TSs present formidable challenges for experimental characterization of protein energy landscape. The advent of single-molecule techniques has made it possible to probe molecules one at a time and visualize their energy landscapes^4–7^. Molecular dynamics simulations have proven to be an extremely useful complement, providing high resolution atomistic level structural insights of processes probed by the single-molecule techniques^8–11^.

Metalloproteins or proteins with metal as a cofactor make up one-third of all known proteins^12^. Despite representing a significant fraction of the proteome, studies of metalloprotein dynamics and folding/unfolding transitions remain scarce^13,14^ and assessing the effect of metal cofactors on protein stability remains a fundamental challenge in the field. Single-molecule force spectroscopy (SMFS) and steered molecules dynamics (SMD) have previously been used to study metalloproteins and probe the effect of metal on the mechanical stability of proteins. Metalloproteins which have been studied include sodium/proton antiporter^15^, GB1 with engineered metal-binding sites^16^, calmodulin^3^, cadherin^17^, rubredoxin^18^, betaine symporter^19^, crystallin^20^, and cupredoxins^21^. The previous studies found diverse effects of the bound metal on the protein energy landscape. For instance, while metal-binding increased the mechanical stability of sodium/proton antiporter^15^, rubredoxin^18^, and crystallin^20^, the bound metal added new pathways for cadherin^17^ and rigidified betaine symporter^19^. Studies on GB1 with engineered metal-binding sites on the protein surface demonstrated how to map the mechanical unfolding transition state. The metal-binding sites that are positioned at the transition state enhance the mechanical stability of the protein whereas the sites away from it do not affect the mechanical stability^16^. In the case of azurin and plastocyanin, Beedle *et al*.^21^ reported complex unfolding features comprising of 2-state and 3-state pathways. The 3-state pathways were stochastic wherein the unravelling of the protein occurred from either termini. The rupture of intermediates in the 3-state pathways has been attributed to the breaking of Cu-S_cys_ and Cu-N_His_ bonds. However, since the authors investigated a mixture of apo- and copper-bound (holo) azurin proteins, the effects induced by copper-binding on the protein unfolding energy landscape could not be directly ascertained. The authors interpreted their results using a model wherein the 2-state pathway was assigned to the apo-protein and the 3-state and 4-state pathways were attributed to the holo-azurin^21^.

Azurin, found in the periplasmic space of gram negative bacteria *Pseudomonas aeruginosa*, is a small 128-residue protein from the cupredoxin family^22^. It has a β-barrel structure, with 8 β-strands arranged in a double-wound Greek-key topology and one α-helix^22^. The structure contains a native disulfide bond between Cys3 and Cys26. Azurin is a metalloprotein with a type-I copper centre exhibiting a trigonal bipyramidal geometry with a central copper bound to two histidine imidazoles (His46 and His117), one cysteine thiolate (Cys112), two weaker axial ligands, sulphur of methionine (Met121) and carbonyl of glycine (Gly45)^23^. Proteins containing type-I copper centre exhibit bright colors in their absorption spectra. Azurin, in particular, shows a strong absorption band with maximum around 625 nm due to S(cys)→Cu^2+^ ligand-to-metal charge transfer transition^24^. The exact function of azurin and the role of copper are not known, although *in vitro* studies suggest that azurin may function as a redox shuttle, facilitating electron transfer in denitrification and/or respiration chains^25,26^. Azurin is an interesting metalloprotein system, where copper-binding does not change the protein structure^22,23^. It appears that the polypeptide fold of apo-azurin has a preformed copper binding site and the binding of copper induces very little change in both, the positions of residues at the metal binding site, and the overall structure of protein (see Fig. S1)^26^. Despite the negligible perturbation in protein structure, we demonstrate using SMFS and SMD that the copper ion (Cu^2+^) alters the unfolding energy landscape of azurin.

To better understand the full complexity of mechanical unfolding pathways of azurin, and the effect of the copper on metalloprotein stability, it is necessary to directly compare protein unfolding in the presence and absence of bound copper. Here, we prepare individual samples of apo- and holo-azurin polyproteins as verified using absorption, steady-state fluorescence and time-resolved fluorescence spectroscopy. Through a study of the mechanical unfolding of apo- and holo-proteins separately using SMFS measurements and SMD simulations, we directly probe the copper-dependent changes in the mechanical unfolding pathways of azurin. We show that apo-azurin unfolds through both 2-state and 3-state pathways, wherein the intermediate in the latter case ruptures through a structurally collective TS. In contrast, holo-azurin unfolds through 2-state, 3-state, and 4-state pathways. While copper-binding does not change the first TS, it makes the protein unravel through different intermediates. The first intermediate unfolds *via* a structurally more localized TS relative to that for the apo-azurin intermediate. In the 4-state unfolding pathway, a second intermediate with copper-sequestered β-strands is formed, which then unravels to produce the third TS leading to complete unfolding of the protein.

## Results

### Apo-azurin unfolds *via* 2-state and 3-state pathways

**Figure 1:**
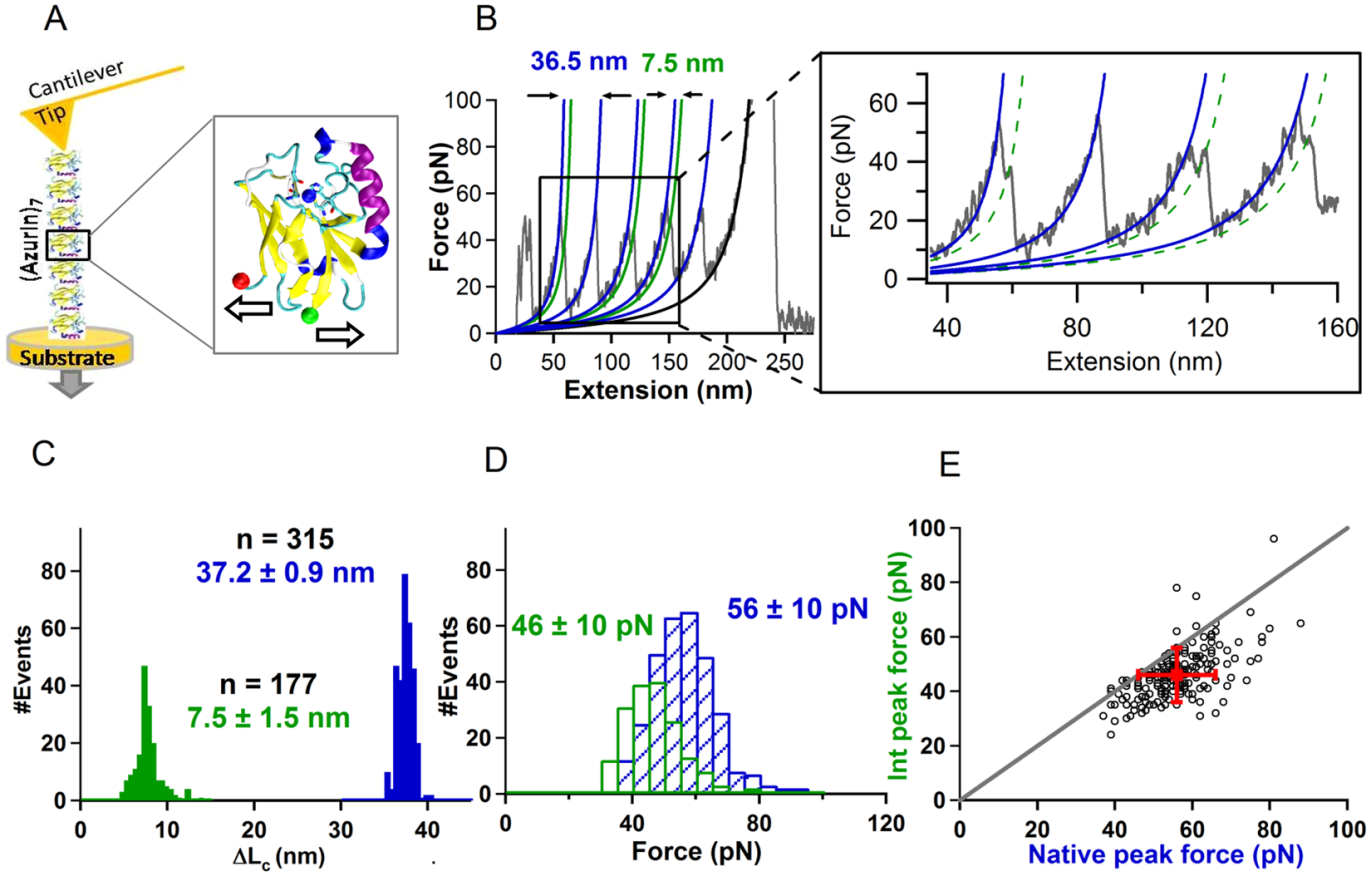
Mechanical unfolding of apo-azurin. (**A**) (*On left*) Schematic representation of SMFS experiment on azurin polyprotein. (*On right*) Structure of holo-azurin (PDB code: 4AZU). (**B**) A representative force-versus-extension (FX) trace of apo-azurin, pulled at 400 nm/s, showing parallel unfolding pathways. Zoomed-in panel shows the trace in more detail. Worm-like chain (WLC) fits to the force peaks of native state, N_apo_ (blue) and intermediate, I_apo_ (green) are shown. More FX data is given in Fig. S8. (**C**) Histogram of the ΔL_c_ of the unfolding of azurin from N_apo_ to U_apo_ (blue) and from N_apo_ to I_apo_ (green). (**D**) Histogram of unfolding force of N_apo_ (blue) and I_apo_ (green). Errors are SD. (**E**) Scatterogram of rupture forces of I_apo_ vs N_apo_ for FX traces showing intermediate. Error bars are SD.

To study the mechanical properties of azurin using SMFS, we followed the chimeric polyprotein approach. Polyproteins provide unambiguous mechanical fingerprints in SMFS experiments^5,6,27–30^. We synthesized a heptamer azurin chain, wherein seven azurins are covalently attached to each other, through N-C termini, using protein engineering tools (see Materials and Methods). Steady-state fluorescence and time-resolved fluorescence spectroscopy data confirmed that the biophysical properties of azurin were unchanged in the heptamer form (Figs S2–S3 and Table S1). SMFS experimental data on the heptamers of apo-azurin is shown in Fig. 1 (see also Fig. S8). A representative force-versus-extension (FX) trace of apo-azurin showing a saw-tooth pattern of unfolding force peaks is given in Fig. 1B. Azurin molecules in the heptamer unfolded with a total contour length change of ΔL_c_ = 37.2 ± 0.9 nm as determined by worm-like-chain (WLC) model fits to the FX data (Fig. 1B,C). This is consistent with the expected ΔL_c_ ~ 36.4 nm [(128–23) aa*0.36 nm/aa-1.4 nm] of the protein with a folded protein end-to-end length of 1.4 nm (calculated from the 4AZU PDB structure) and with a disulfide bond (Cys3-Cys26) sequestering 23 residues. In the trace shown in Fig. 1B, three azurins (1, 3, and 4 from left in the saw-tooth pattern) have two force peaks indicating that these azurin molecules unfolded through the intermediate I_apo_. The ΔL_c_ from the native state (N_apo_) to the intermediate (I_apo_) was determined to be 7.5 ± 1.5 nm (Fig. 1B,C). The mechanical rupture forces of N_apo_ and I_apo_ were 56 ± 10 pN and 46 ± 10 pN, respectively (Fig. 1D). A correlation analysis showed that the rupture force of I_apo_ was consistently lower than the rupture force of the corresponding N_apo_ (Fig. 1E). Analysis of the apo-azurin FX traces revealed that N_apo_ unfolded via a parallel 3-state unfolding pathway in 56 ± 3% of the events. The errors were estimated using bootstrap procedure as described in Methods. To see if the 3-state unfolding flux is dependent on the pulling speed, we varied the pulling speed by two orders of magnitude (40–2000 nm/s) and observed that the 3-state flux with an intermediate varied from 32–85% (see Table S7). It is possible that the metastable intermediate is resolved in the unfolding traces more efficiently at higher pulling speeds and its detection at low pulling speeds is limited by the resolution of the instrumentation.

### SMD simulations reveal both structurally localized and collective TSs for apo-azurin unfolding

**Figure 2:**
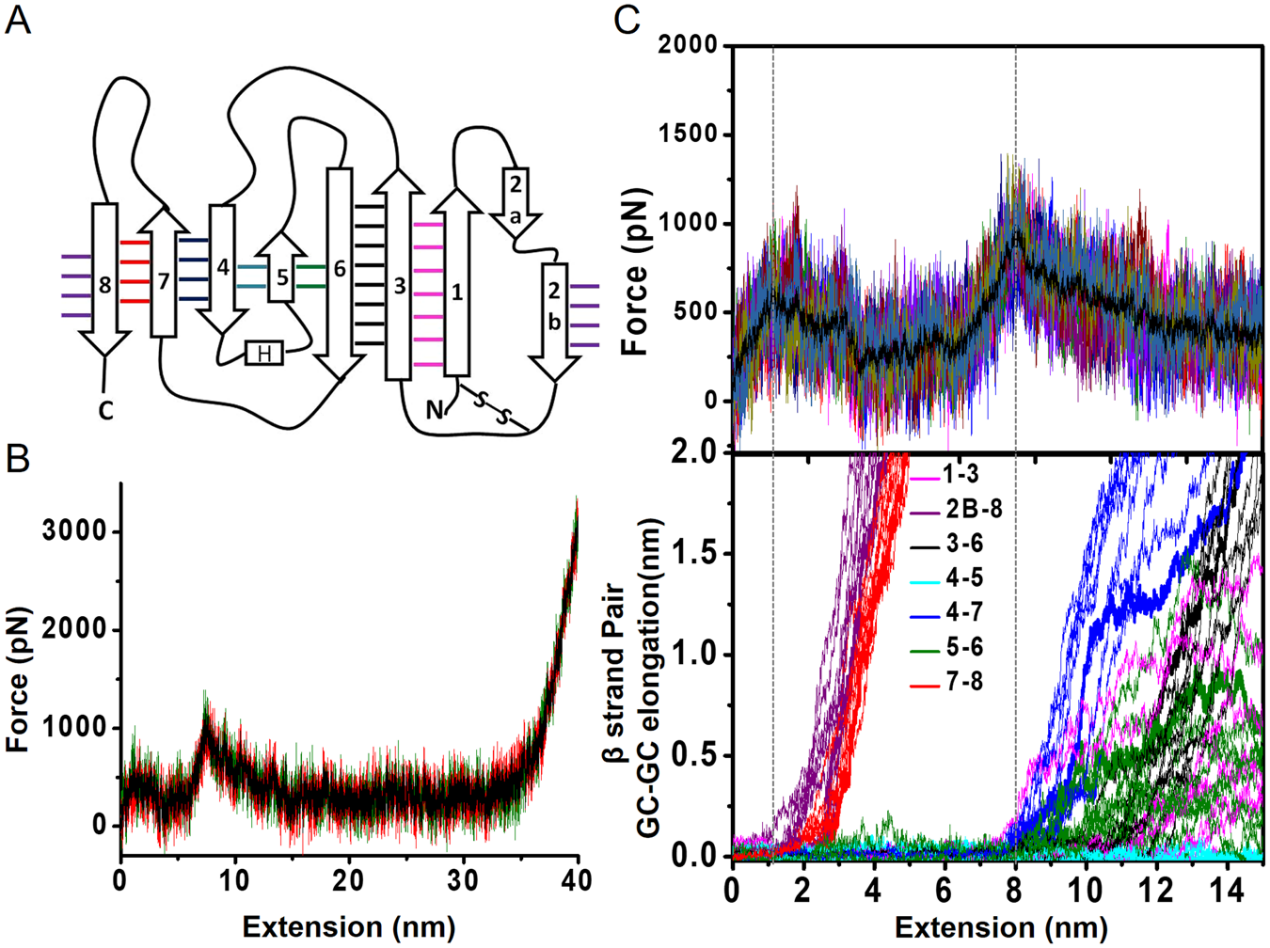
Steered molecular dynamics (SMD) simulation of apo-azurin. (**A**) 2D topology of apo-azurin. See Table S5 for β-strand assignment. (**B**) Three SMD force-versus-extension (FX) traces showing two force peaks of apo-azurin in C-terminal pulling simulations. (**C**) (*Top*) Overlay of 10 FX traces showing two TSs obtained by using different initial structures from NVT equilibrium of 100 ns. An average FX trace is also shown (solid black line). (*Bottom*) Geometric centres (GCs) of β-strands are calculated using the C_α_ atoms of the constituting residues. GC-GC distance change of adjacent β-strands as a function of unfolding extension during C-terminal pulling of apo-azurin from 10 trajectories. Data for N-terminal pulling simulations is shown in Fig. S9.

We carried out multiple *in silico* constant velocity pulling simulations on fully solvated and atomistic equilibrated models of apo-azurin structures (see Materials and Methods). In these simulations the C_α_ atom of one terminal residue was held fixed while pulling the C_α_ atom of the other terminal residue to unfold the protein. We carried out both C-terminal as well as N-terminal pulling simulations (Figs 2 and S9). From the simulations we extracted FX traces which could be compared with those from the experiments. The FX traces obtained from simulations clearly show two force peaks, confirming the existence of the I_apo_ seen in experiments (Fig. 2). The close overlap of the FX traces from multiple simulations indicated reproducible results which capture the unfolding behaviour of the protein (Fig. 2B,C). We carried out a detailed analysis of structures underlying each FX trace from SMD simulations to extract unfolding pathways of apo-azurin and associated TS structures. We analyzed the sequence of unfolding events in terms of changes in the distance between geometric centres (GCs) of adjacent β-strands in the azurin topology (Figs 2A,C, S9 and Table S5). We find that β2B–β8 and β7–β8 contacts are ruptured at the first TS, followed by the unravelling of β8. Thus, the structural changes leading to the first TS are localized to β8 and its flanking strands. In contrast, the unfolding at the second TS is due to a collective rupture of interactions between all remaining β-strand pairs (β1–β3, β3–β6, β4–β7 and β5–β6). At the second TS, β4–β7 pair ruptured (~1.5 nm GC-GC distance change for a 2 nm change in molecular extension) prior to the other β-strand pairs (<1.0 nm GC-GC distance change for the same 2 nm change in molecular extension) in 8 out of 10 instances. In the remaining cases, β1−β3 pair ruptured (~0.5 nm GC-GC distance change) prior to the response of β4−β7, which followed the response of other pairs. These simulation results suggest a structurally collective TS at the second unfolding force peak, wherein the β-strand interactions ruptured at the TS are spread over a large part of the protein.

### Copper-binding shifts the position of the second TS and number of intermediates during azurin unfolding

**Figure 3:**
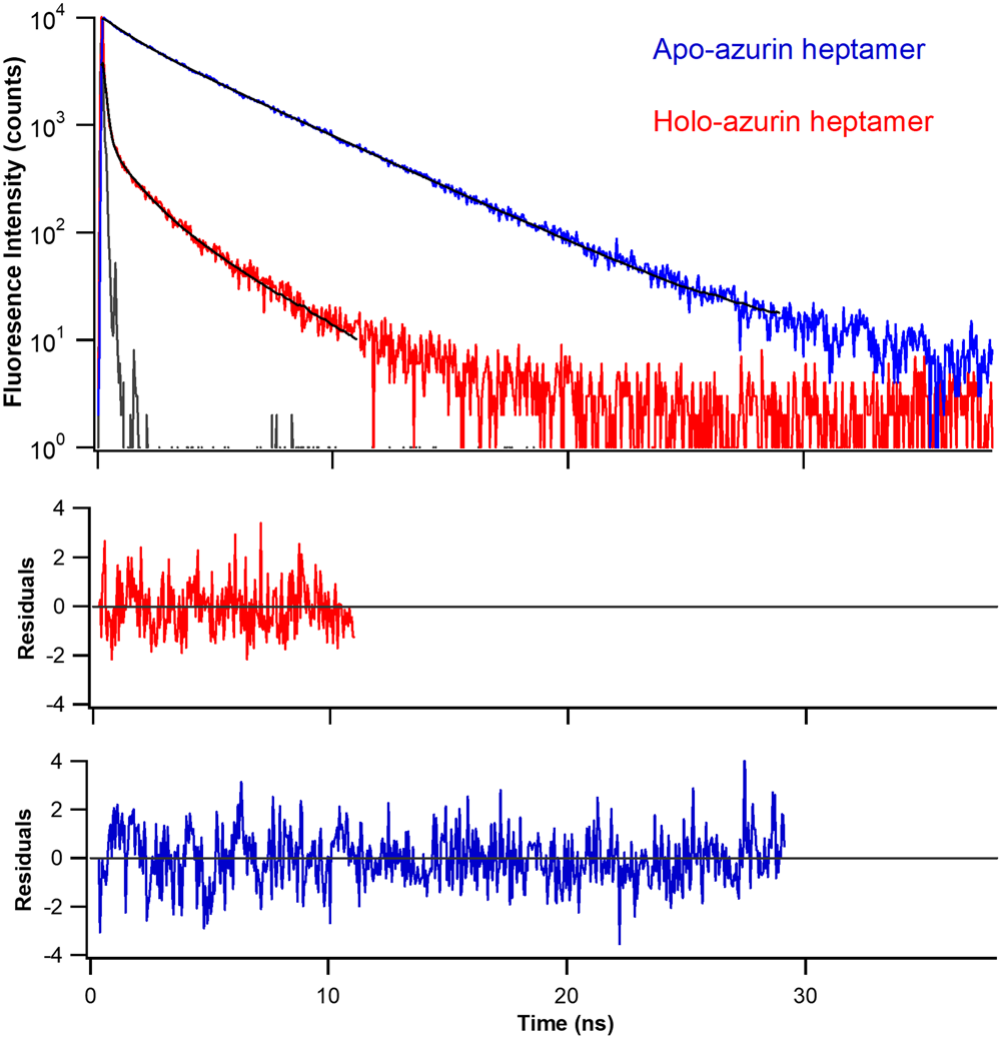
Time-resolved fluorescence intensity of heptamers of apo- and holo-azurins. Trp48 in the azurins is excited with 295 nm light. The fluorescence emission was collected at 310 nm. Instrument response function (grey solid-line) and multi-exponential fits to experimental data (black solid-lines) are shown. Residuals are shown in the bottom panels. The parameters of the fits are given in Table S1.

To obtain direct evidence of the effect of copper on the mechanical properties of azurin, we carried out SMFS experiments on Cu^2+^-bound azurin heptamer (holo-azurin). We prepared holo-azurin in buffered solution containing 1 mM CuSO_4_ to ensure that azurin was present in the copper-bound form and confirmed it independently by well-established spectroscopic methods (see Materials and Methods, Figs 3, S2 and Table S1). The absorption spectrum of the holo-azurin heptamer has a charge transfer band centered at 628 nm, which confirms the copper binding (Fig. S2). This band is absent in the apo-azurin heptamer indicating the absence of copper. We have also carried out steady-state fluorescence spectroscopy which shows that the spectral shape of fluorescence emission of Trp48 in azurin is unchanged between monomer and heptamer forms. This result indicates that the apo-azurin structure is preserved in the heptamer form, as the binding of copper is expected to quench the fluorescence emission of Trp48^31^. We further analysed the decrease in Trp48 fluorescence by more sensitive time-resolved fluorescence spectroscopy measurements which showed a decrease in the mean fluorescence lifetime (τ_m_) from 3.45 ns to 0.30 ns due to copper binding (Fig. 3). Moreover, these measured lifetimes of heptamers are comparable with that of the azurin (monomer) (see Table S1). The properties of heptamer are in concurrence with that of the monomer and that of the literature confirming that the apo-protein is free of copper and holo-form contains bound copper. Once, the holo-form heptamer was confirmed with copper binding, we proceeded to SMFS experiments.

**Figure 4:**
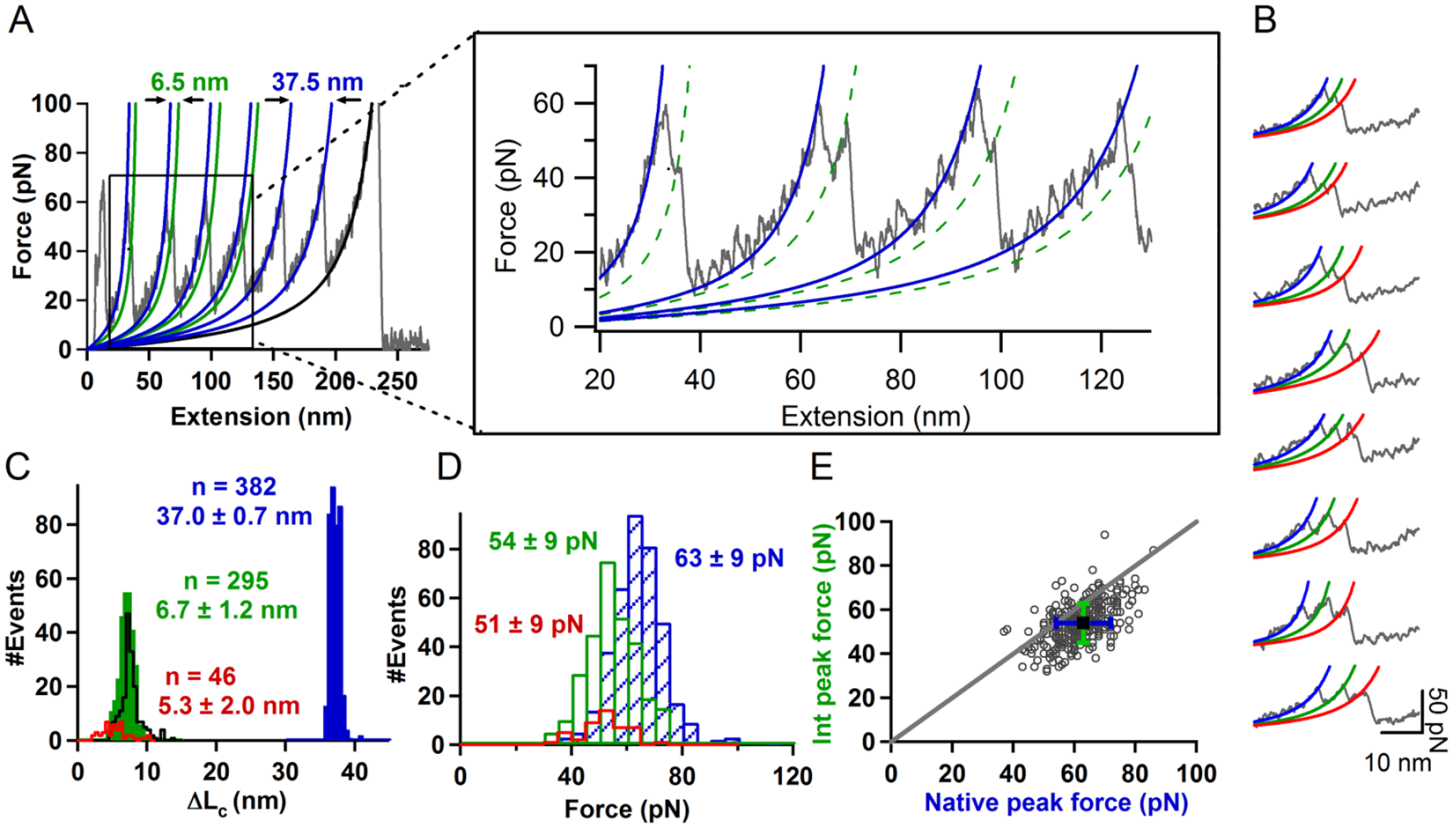
Mechanical unfolding of holo-azurin. (**A**) Representative FX trace of holo-azurin showing intermediate. Zoomed-in panel shows the trace in more detail. WLC fits are shown for the unravelling of native state, N_holo_ (blue) and intermediate, I_holo_ (green). More FX data is given in Fig. S10 in the Supplementary Material. (**B**) Unfolding events of holo-azurin that followed 4-state pathways: N_holo_ to I_holo_ to I’_holo_ to U_holo_. WLC fits are shown for native state, N_holo_ (blue), intermediate, I_holo_ (green), second intermediate, I’_holo_ (red). (**C**) Histogram of the total change in contour length (ΔL_c_) of unfolding of azurin from N_holo_ to U_holo_ (blue), N_holo_ to I_holo_ (green), and I_holo_ to I’_holo_ (red). Errors are SD. ΔL_c_ histogram for N_apo_ to U_apo_ (black) is also shown. (**D**) Histogram of unfolding force of azurin from rupture of N_holo_ (blue), I_holo_ (green), and I’_holo_ (red). Errors are SD. (**E**) Scatterogram of I_holo_ peak force vs N_holo_ peak force for unfolding events showing intermediate (I_holo_). Error bars are SD.

The SMFS pulling experiments on holo-azurin showed that the native state (N_holo_) unfolded with a total ΔL_c_ = 37.0 ± 0.7 nm analogous to N_apo_ (Figs 4 and S10). We quantified the unfolding pathways captured by the experimental traces by estimating the percentage flux and associated errors. Errors were estimated using a bootstrap procedure described in Methods. While 77 ± 2% of molecules of holo-azurin exhibited an intermediate (I_holo_), a small fraction (16 ± 2%) of these molecules further unfolded through a second intermediate (I’_holo_), in a 4-state manner with three rupture force peaks (Fig. 4A,B). Such 4-state unfolding was rare in the case of apo-azurin (3% flux). The 2-state unfolding pathway flux for holo-azurin was lower (23 ± 2%) as compared to that for apo-azurin (44 ± 3%). As in the case of apo-azurin, to see if the 3-state unfolding flux was dependent on the pulling speed, we varied the pulling speed (40–2000 nm/s) and observed that the 3-state flux varied between 39–86% (see Table S8). Notably, both apo- and holo-azurins show parallel pathways at all pulling speeds. The rupture forces of N_holo_, I_holo_, and I’_holo_ were 63 ± 9 pN, 54 ± 9 pN and 51 ± 9 pN, respectively (Fig. 4D). Correlation analysis of the rupture forces showed that I_holo_ is mechanically weaker compared to N_holo_, as also seen in the case of apo-azurin (Fig. 4E). Interestingly, the ΔL_c_ from N_holo_ to I_holo_ (6.7 ± 1.2 nm) is about ~1 nm shorter than that for apo-azurin. The statistical *p*-value for the difference in ΔL_c_ of the apo- and holo-azurin intermediates is <0.00001, implying that the observed ΔL_c_ difference is significant. The ΔL_c_ of the second intermediate from I_holo_ to I’_holo_ is 5.3 ± 2.0 nm. The large spread in the contour lengths suggests heterogeneity in the unfolding pathways which can arise, for instance, due to the rupture of different interactions in the β1-β3-β6 motif. The structural changes behind the contrasting features in the mechanical properties of apo- and holo-azurins are further revealed in SMD simulations.

### Presence of copper-coordination sphere sequesters β4**–**β7 pair from rupture and changes the unfolding pathway of azurin

**Figure 5:**
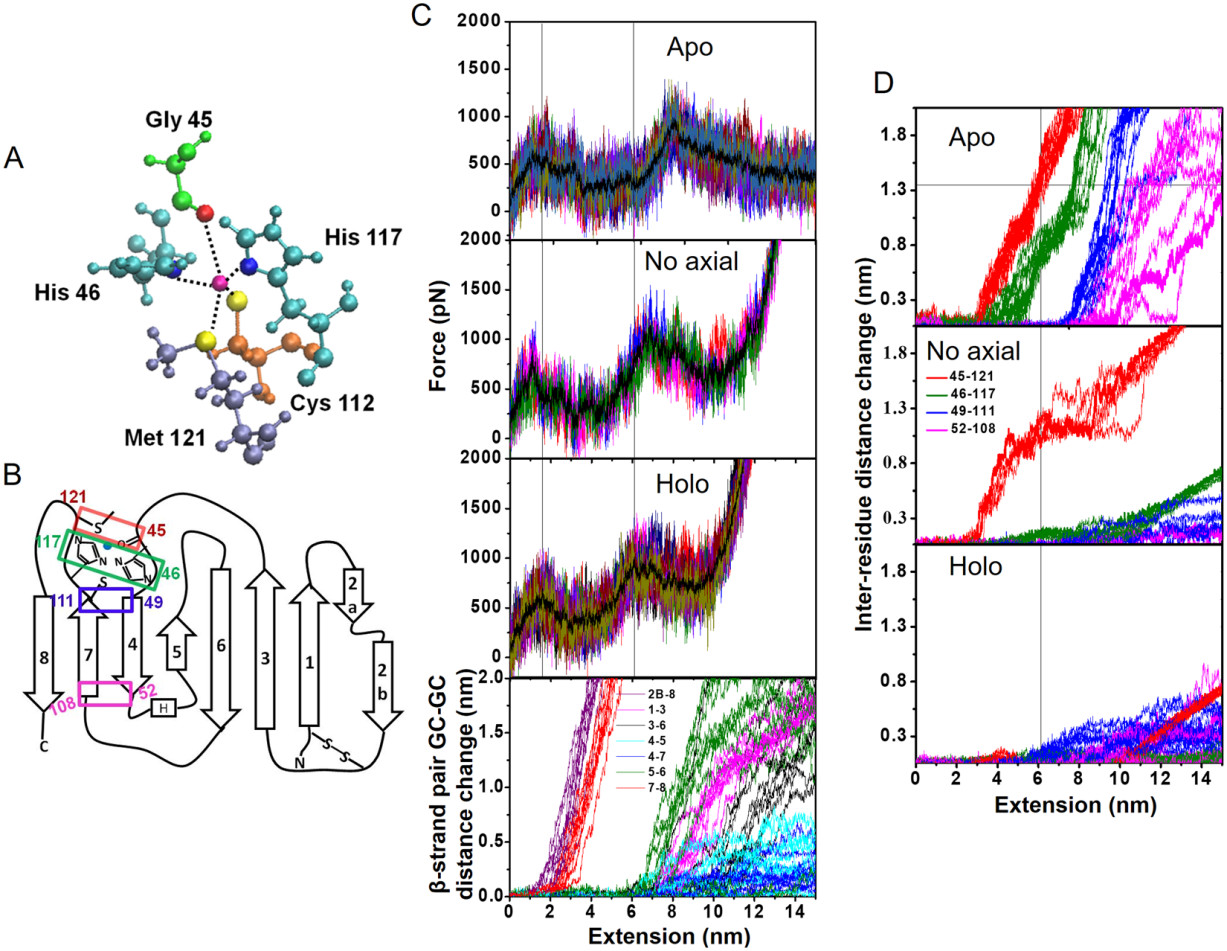
Comparison of unfolding of apo- and holo-azurins. (**A**) Copper coordination sphere in holo-azurin. (**B**) 2D topology showing inter-residue separations of the copper coordination sphere and those of the β4–β7 strand pair. (**C**) (*Top three panels*) Simulated FX traces (10 simulations) of three different forms of apo-azurin, ‘no axial’ holo-azurin, and holo-azurin. (*Bottom*) GC-GC distance change of adjacent β-strands as a function of C-terminal pulling of holo-azurin. All copper-ligand coordination bonds in holo-azurin and three planar equatorial copper-ligand bonds in ‘no axial’ holo-azurin were kept at a spring constant 25 kcal/mol/Å^2^ (see text for more details). Comparison of peak positions in FX-traces (reference dotted lines show peak positions for holo-azurin) shows that the position of the second TS peak is shifted to shorter extensions due to the presence of copper in accord with experimentally observed changes in ΔL_c_ (Fig. 4B). Corresponding peak positions and inter-peak separations are reported in Table S6. (**D**) Inter-residue distance (C_α_-C_α_ separation) change for residue pairs shown in Fig. 5B during unravelling of different forms of azurin. Thin vertical line represents the position of the second TS for holo-azurin. Thin horizontal line represents the separation of residue pair Gly45-Met121 in apo-azurin. Intersection of these two thin lines explains the larger ΔL_c_ for the I_apo_ relative to that of I_holo_. Note that for the ‘no axial’ model, Gly45-Met121 pair separates less relative to apo-azurin thereby producing an intermediate ΔL_c_ between that of apo- and holo-azurins. Corresponding GC-GC distance change comparisons are provided in Fig. S13.

For SMD simulations on holo-azurin, we considered a model for the copper coordination sphere which included non-bonding interactions (electrostatic and van der Waals potential terms) and a description of copper-ligand coordination bonds as harmonic springs with equal spring constants (see Materials and Methods). The representation of a metal-coordination sphere by a classical potential is a gross oversimplification and may introduce modelling artifacts in the SMD generated FX traces. Therefore, in order to obtain unbiased insights, we carried out systematic tests by varying the coordination bond spring constant over two orders of magnitude (1–100 kcal/mol/Å^2^), spanning conventional bond strength ranges exhibited by hydrogen bonds and covalent bonds. Multiple SMD simulations were performed at each spring constant value (Fig. S11). The results showed that for spring constant values <25 kcal/mol/Å^2^, FX traces resembled those of apo-azurin. Figure 5 shows results for C-terminal SMD pulling simulations with spring constant 25 kcal/mol/Å^2^ (similar results were obtained for N-terminal pulling simulations and higher spring constant values; Figs S11 and S12). These FX traces of holo-azurin produced two force peaks within a 10 nm extension (Fig. 5C) confirming the presence of I_holo_ observed in experiments. While the first TS peak position is unaltered with respect to that of the apo-azurin simulation trajectory; the peak position of the second TS is shifted to lower molecular extensions (Fig. 5C). Notably, the average peak separation for the holo-azurin traces (4.5 nm) is ~1.7 nm shorter than that of apo-azurin simulation, in accord with experimentally observed shortening of ΔL_c_ between apo- and holo-forms (Fig. 4C). Formulating the sequence of unfolding events in terms of distance changes between GC of adjacent β-strands reveals that the different peak separations for apo- and holo-azurins reflect different unfolding pathways for the two proteins (Fig. 5C). At the second TS, copper sequesters β4–β7 pair from rupture because of the constraints imposed by the copper-ligand bonds. The unfolding of I_holo_ is due to a collective rupture of interactions between the β-strands pairs: β1–β3, β3–β6, β4–β5 and β5–β6. In almost all traces, the β1–β3 and β5–β6 pairs ruptured (GC-GC distance changed by ~1 nm for a 4 nm change in molecular extension) prior to the other β-strand pairs (<0.5 nm GC-GC distance change for the same 4 nm change in molecular extension). In one instance, β3–β6 pair ruptured prior to the response of β1–β3 (Fig. 5C). While the second TS of holo-azurin is still collective, involving many interactions spread over the protein, it is structurally more localized than that for apo-azurin as it excludes the β4–β7 pair.

To highlight the imprint of the copper coordination sphere in the FX trace of holo-azurin, we further carried out SMD simulations constraining only the equatorial copper-ligand bonds (i.e., Cu–Cys112:S, Cu–His117:N and Cu–His46:N) and no harmonic constraints on the axial bonds (i.e., Cu–Gly45:O, Cu–Met121:S). In these simulations, the second TS occurred at extensions intermediate to that seen for apo-azurin, and holo-azurin with all copper-ligand bonds constrained. From our analysis (Figs 5C,D, and S13), the shift in the second TS of holo-azurin relative to that for apo-azurin can be understood as follows. In the case of apo-azurin, with no metal coordination sphere, the protein is free to unravel after the first TS till the end of β7 (i.e., up to Cys112). In contrast, in holo-azurin with ‘no axial’ copper coordination, the protein extension is curtailed to His117. For holo-azurin with full copper coordination, the extension is further curtailed to Met121. Figure 5D compares the sequence of residue separations (C_α_–C_α_ distance change) in the immediate vicinity of the copper binding site and β4−β7 along the unfolding pathways of different forms of azurin (see Fig. S13). From Fig. 5D (thin-lines), it is clear that the change in separation of the Gly45-Met121 pair at the second TS between apo- and holo-azurins accounts for the ~ 1 nm shortening of ΔL_c_ between apo- to holo-forms observed in experiments (Fig. 4C).

### Controlled rupture of copper-coordination bonds in holo-azurin and the manifestation of third TS

**Figure 6:**
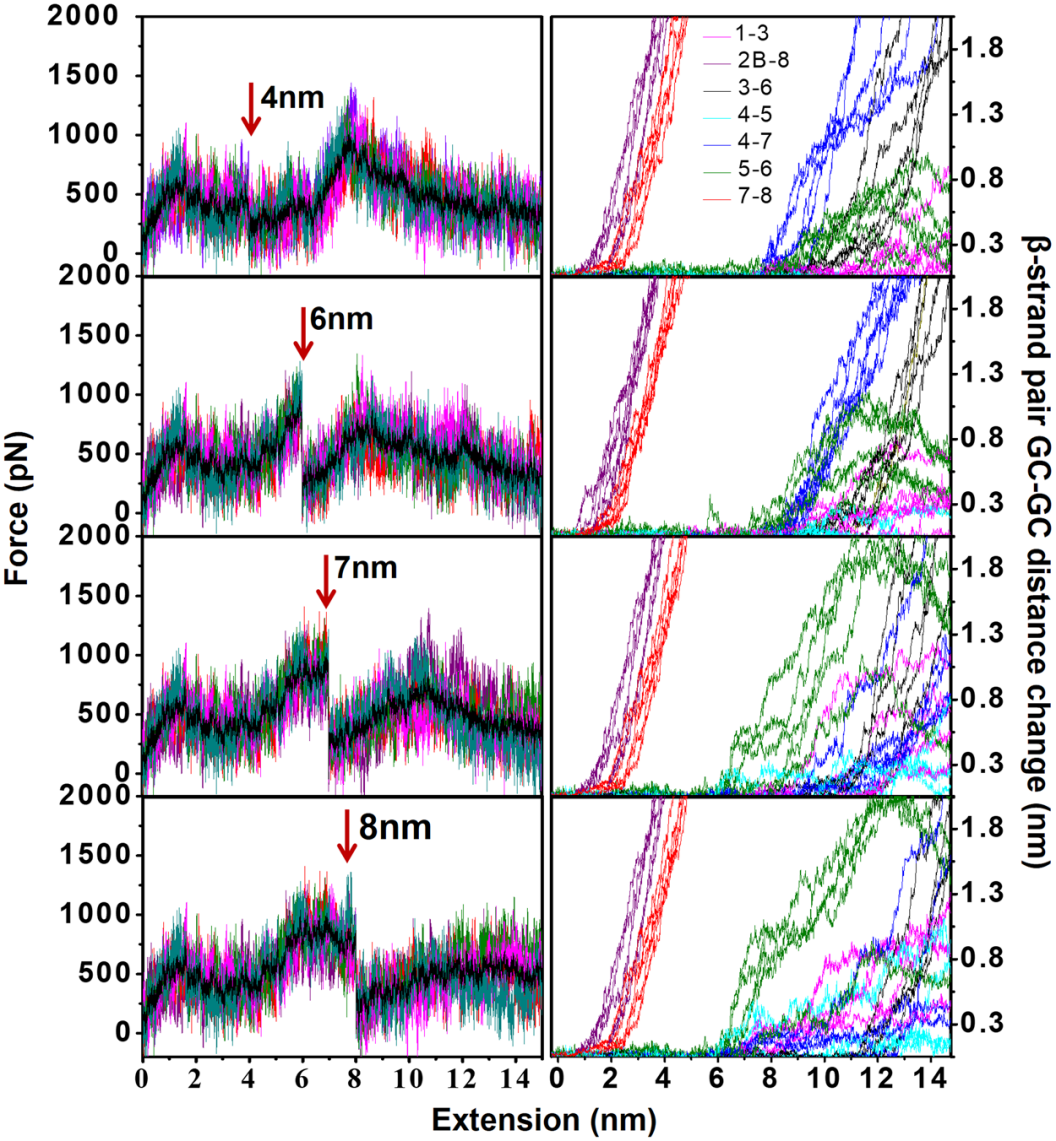
Controlled rupture of copper-ligand coordination sphere at different positions along the unfolding trajectory. (*On left*) Simulated FX traces of holo-azurin unfolding with all five copper-ligand bonds ruptured by removing harmonic constraints at different positions along the extension: 4 nm (i.e., before the second TS), 6, 7 and 8 nm (i.e., at the second TS). For each rupture condition 5 FX traces and their average (solid black line) are shown. Arrows show the position where the copper-ligands were ruptured. (*On right*) Corresponding GC-GC distance changes of β-strand pairs with molecular extension.

Unlike the case of apo-azurin, the FX traces from our classical holo-azurin SMD simulations do not capture the complete unravelling of azurin (Figs 2B and 5C) due to the imposition of harmonic constraints on the copper coordination sphere. We therefore carried out new simulations wherein the harmonic constraints on the copper-ligand bonds were removed at different extensions along the holo-azurin unfolding pathway (Fig. 6). From the FX traces in Fig. 6 it is clear that a rupture of the copper coordination sphere at 4 nm (i.e., before the second TS) produces a FX trace identical to that seen for apo-azurin simulations (Fig. 2C). This result highlights the selective imprint of the copper on the second TS of holo-azurin. When the rupture of the copper coordination sphere is delayed past the second TS a broad second force peak is produced at an extension ~1.7 nm shorter than that for apo-azurin (Fig. 5C). If the copper coordination sphere rupture occurs at or beyond the second TS, our simulations predict (bottom three panels in Fig. 6) a third force peak corresponding to the separation of the β4−β7 pair whose position depends on the extension at which the copper coordination sphere breaks. Rupture at 6, 7 and 8 nm extensions, where β4–β5 and β5–β6 elongated to different extents, produces a third force peak ~2.7, 4.7, and 5.8 nm away from the second force peak respectively (Table S6 in the Supplementary Material). In Fig. 6, the GC-GC distance change plots for the simulated FX traces attribute the third force peak to the β4–β7 separation (Fig. 6). We note that in the simulations presented in this section, all copper-ligand bonds rupture simultaneously, producing a sharp, almost vertical drop in force with extension in all FX traces. Since such features are absent in the experimental FX traces, we reason that the copper-ligand bonds must rupture sequentially. Our simulations do not resolve contributions from copper-ligand bonds to the third TS. The results presented here show that the copper-ligand bonds have to rupture at or after the second TS in the holo-azurin unfolding pathway. Rupture of the copper-ligand sphere along with the sequestered β4–β7 pair can contribute to the formation of a third TS. Interestingly, a small population of I_holo_ (~16%) in experiments showed a third TS leading to I’_holo_.

## Discussion

**Figure 7:**
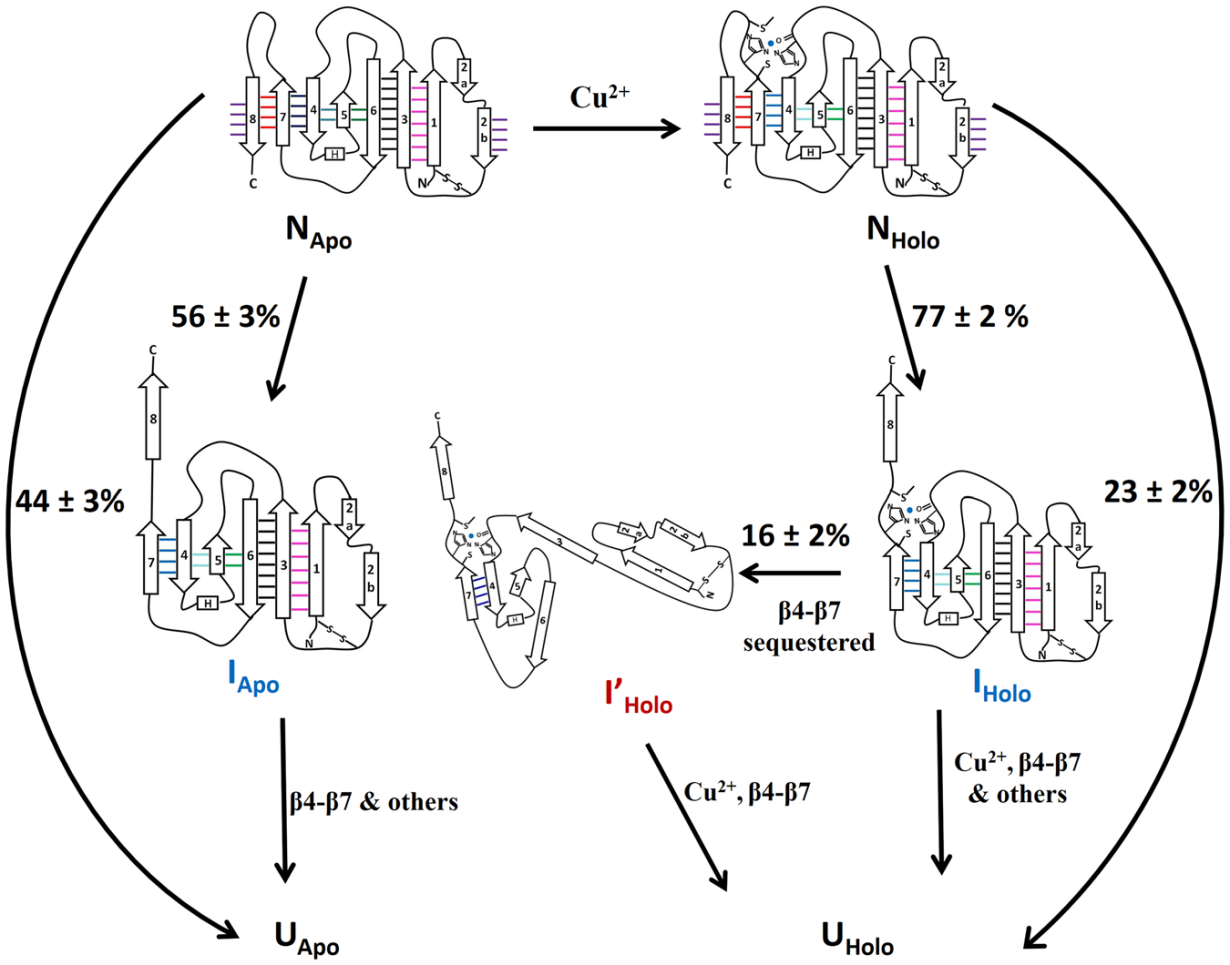
Schematic showing the unfolding mechanism of apo- and holo-azurins from their native states (N_apo_ and N_holo_) to the unfolded states (U_apo_ and U_holo_). The flux data through the parallel mechanical pathways was obtained through SMFS experiments and the structural information on the sequence of β-strand rupture and unravelling through SMD simulations. Apo-azurin follows both 2-state and 3-state pathways, whereas holo-azurin follows 2-state, 3-state and 4-state pathways. The intermediates on the unfolding pathways of apo- and holo-azurin are different.

Our view of the unfolding pathways of apo- and holo-azurins derived from single-molecule mechanochemistry experiments and non-equilibrium MD simulations is depicted as a schematic in Fig. 7. In 56 ± 2% of unfolding events, apo-azurin (N_apo_) unravels *via* a structurally localized TS with the rupture of interactions between β2B–β8 and β7–β8, to a well-defined intermediate (I_apo_). In contrast, I_apo_ unfolds through a structurally collective TS due to the rupture of interactions spread over the remaining protein structure. Ensemble unfolding experiments have classified TSs into polarized (or local) and diffuse TSs mainly based on ϕ-value analysis^32–38^. A local TS is defined as one where the high ϕ-value residues are clustered over a small region of a protein^39,40^. In contrast, for diffuse TS, most of the residues of the protein have similar ϕ-values^41,42^. The structurally localized and collective TSs captured in azurin unfolding pathways are analogous to the picture of local and diffuse TSs based on ϕ-value analysis. To the best of our knowledge, our study is the first to provide fingerprints of structurally collective TSs along the mechanical unfolding pathways of proteins. Upon copper-binding, the first TS of holo-azurin (N_holo_) remains unchanged from that of apo-azurin, and leads to I_holo_ with a copper-coordination sphere. Further unravelling of the protein differs from that in N_apo_, with elongation curtailed by ~1 nm relative to that for apo-azurin due to constraints imposed by the copper coordination sphere. As the copper coordination sequesters β4−β7, it has to rupture first for β4−β7 elongation. If the copper coordination of I_holo_ ruptures early at the second TS, then the β4−β7 rupture and subsequent extension is only separated by ~2–3 nm from the second TS position and cannot be resolved as a separate force peak in SMFS experiments. Thus, early copper rupture at the second TS results in 3-state pathways as observed for 84% of I_holo_ population. However, if the rupture of the copper-coordination sphere is delayed then I_holo_ unfolds to I’_holo_ through a structurally more localized second TS (relative to that of apo-azurin) with copper sequestering the β4−β7 strand pair. Subsequent rupture of the copper coordination sphere along with separation of β4–β7 pair through a structurally localized third TS results in a 4-state pathway as observed for 16% of I_holo_ population in SMFS experiments. Diffuse TSs in protein folding have earlier been reported in ensemble experiments^38^. However, in SMFS experiments, the canonical view is that rupture of interactions at the TSs is structurally local in nature. For example, mechanical unfolding of titin I27 or ubiquitin has been attributed to the rupture of H-bonds between terminal β-strands in^43,44^.

Earlier studies by Beedle *et al*.^21^ on the mechanical unfolding of a mixture of apo- and holo-azurin polyproteins using constant-velocity pulling experiments and constant-force SMD simulations showed complex unfolding pathways with multiple intermediates. The authors observed 20% molecules unfolding through a 2-state pathway, which they attributed to be coming from apo-azurin molecules. The remaining 80% population showed 3-state and 4-state pathways. The 3-state pathways were stochastic and the unfolding originated either from the N-terminus or from the C-terminus, which is based on their observation of two different unfolding contour length values for the intermediates. In 4-state pathways, both of these intermediates were observed one after the other before complete unfolding. Interestingly, their constant-force SMD simulations suggested that the majority of the population (90%) unfolded from the C-terminus and the remaining population unfolded either from the N-terminus or from both the termini^21^. Beedle *et al*.^21^ also attributed the rupture of intermediates to the breaking of Cu-S_cys112_ and Cu-N_His117_ bonds. Our study differs from that by Beedle *et al*.^21^ in two crucial aspects. First, we carry out independent SMFS measurements on apo- and holo-azurin polyprotein samples. Secondly, we carry out constant-velocity SMD simulations which directly mimic the experiments. Though some of the aspects of complex unfolding pathways of holo-azurin from Beedle *et al*.^21^ agree with our results, there are also notable differences. Our results show that 2-state pathways are possible for both apo- and holo-azurins. Further, the unfolding of native states always occurred from the C-terminus in our constant velocity SMD simulations. This result is in agreement with our experiments where we observed only one unfolding contour length for the intermediate and the native state along each unfolding pathway. Constant-force simulations by Beedle *et al*.^21^ also show that azurin predominantly unfolds from C-terminus. Most importantly, the copper-binding effects manifest in terms of a subtle 1 nm change in the unfolding contour length of the native state to the intermediate. We stress that the copper induced subtle 1 nm shift of the intermediate could not have been observed without independent measurements on apo- and holo-azurins. Our findings suggest that apo-azurin follows 2-state and 3-state unfolding pathways. Copper-binding, shortens the contour length leading up to the second TS and may produce a third TS (4-state unfolding). This picture differs from the proposal by Beedle *et al*.^21^ that apo-azurin unfolding occurs exclusively *via* a 2-state pathway, whereas holo-azurin unfolds *via* 3-state and 4-state unfolding pathways. Furthermore, Beedle *et al*.^21^ propose that the intermediate force peak observed during azurin unfolding is entirely due to copper-ligand bond rupture. In contrast, we show clearly that apo-azurin also unfolds *via* 3-state pathway, suggesting that the rupture at the second TS observed in holo-azurin by Beedle *et al*.^21^ may not solely be due to the rupture of copper-ligand bonds.

Our systematic SMFS constant-velocity experiments together with constant-velocity SMD simulations of apo- and holo-azurins unambiguously provide the evidence for copper-binding induced changes in the mechanical unfolding pathways of azurin. Copper-binding modulates the unfolding energy landscape specifically at the second TS leading to create parallel unfolding pathways in holo-azurin which differ in the sequence of β-strand separation events and number of intermediates from that in apo-azurin. Such a kinetic partitioning of parallel pathways observed here for single domain apo- and holo-azurins has been reported previously only for multi-domain proteins^5,7,45–49^.

## Conclusions

Our results significantly further our understanding of the effect of metal ions on the unfolding energy landscape and pathways of metalloproteins. Earlier studies on the unfolding of proteins with ligands have highlighted direct changes in the mechanical stability of proteins^17,18,28^. Recently, it was shown for ferridoxin that metal coordination leads to an intermediate^50^. In the present study, however, the mechanical stability of azurin in terms of rupture force remains largely unaltered upon copper binding. Rather, new unfolding pathways are introduced to make the energy landscape of protein more rugged. While previous studies on metalloproteins have revealed the role of metal containing active site and the individual metal-ligand bonds on the mechanical properties of proteins, the proteins were themselves mechanically compliant with the entire mechanical resistance due to the coordination bonds^18,51^. Further studies on other metalloproteins are necessary to establish the generality of the metal-induced sequestering mechanism during the mechanical unfolding of proteins. However, the effect of metal in azurin is complex, creating no major change in the rupture force, yet altering unfolding pathways dramatically in terms of sequence of rupture events as well as intermediates. Our studies add to recent reports of copper-induced changes in mechanochemical reactions within single-molecules^52^. The analytical framework using combined force spectroscopy experiments and SMD simulations presented here should be useful to study metal induced changes to protein energy landscapes of other metalloproteins and enhance our understanding of the roles of metal on protein mechanical stability and conformational flexibility.

Furthermore, azurin is a periplasmic protein. It is synthesized in cytosol and then undergoes translocation into the periplasm. Forced unfolding of proteins prior to their insertion into the narrow pores of protein channels in the membrane is a key step in the translocation process^53,54^. Hence, the mechanical unfolding pathways of azurin and the intermediate states captured here might be important in understanding its translocation process *in vivo*.

## Materials and Methods

### Polyprotein engineering

The azurin gene cloned in pGK22 vector was taken for polyprotein construction^55^. The heptameric construct of azurin was cloned in pQE80L expression vector (Qiagen Valencia, CA) between the restriction endonuclease sites *BamHI* and *HindIII* using the iterative cloning technique described previously^27^.

Azurin-heptamer containing pQE80L plasmid was over-expressed in *E. coli* BL21 (DE3) cells using 1 mM IPTG induction at an optical density (OD_600_) of 0.6 of the cell culture and then incubating at 37 °C for 6 hrs. The cell pellet expressing the apo-protein was resuspended in Tris-HCl (20 mM, pH 7.4) in the presence of 1 X protease inhibitor cocktail (Sigma Aldrich) and lysed by sonication at 4 °C. Lysate obtained after centrifugation (17,000 rpm for 45 min at 4 °C) was incubated with Ni-NTA resin (Roche) for 16 hrs at 4 °C on rotaspin. The resin was washed with Tris-HCl (20 mM, pH 7.4) and Tris-HCl (20 mM, pH 7.4) containing 20 mM imidazole after which azurin heptamer with His_6_–tag at the N-terminus was eluted using Tris-HCl (20 mM, pH 7.4) containing 250 mM imidazole. Apo-protein was further purified using superdex200 columns (GE Healthcare) on Bio-Rad Biologic Duo-Flow FPLC system. Purity of the protein was checked using SDS-PAGE (see Fig. S2).

Holo-azurin heptamer was prepared in 1 mM CuSO_4_ containing Tris-HCl (20 mM, pH 7.4) buffered solution, and copper-binding was confirmed by absorbance at 628 nm (A_628_), steady-state fluorescence, and time-resolved fluorescence spectroscopy (Figs 3, S2 and Table S1)^56^. Purification procedure of azurin monomer is also given in Supplementary Material.

### Spectroscopic characterization of apo- and holo-azurin heptamers

Purity of protein and copper-binding was further confirmed by absorption and fluorescence spectroscopy (see Figs 3 and S2). Fluorescence measurements were carried out at a protein concentration of 10 μM. All the steady-state measurements were carried out on a SPEX fluorolog (T-format) fluorimeter by exciting the protein samples at 295 nm wavelength. The fluorescence emission spectral properties of apo-azurin remained unaltered in the heptamer (Fig. S3).

Time-resolved fluorescence intensity measurements were carried out using a time correlated single photon counting (TCSPC) instrument coupled to a microchannel plate photomultiplier (Model 2809 u; Hamamatsu Corp), as described previously^56^. Time-resolved fluorescence intensity was measured by employing CW-passively mode-locked frequency-doubled Nd:YAG laser (Vanguard, Spectra Physics, USA) driven Rhodamine 6 G dye laser. Dye laser pulses at 590 nm frequency-doubled to 295 nm were used for excitation. The repetition rate of the laser pulses was 4 MHz. Time per channel of the collected time-resolved data was 48.9 ps. The half-width of the instrument response function (IRF) at 295 nm was ~80 ps. The fluorescence emission was collected through a 305 nm cutoff filter followed by a monochromator set at 310 nm. The cutoff filter was used to prevent scattering of the excitation beam from the samples. Fluorescence was collected with the emission polarizer oriented at the magic angle (54.7°) with respect to the excitation polarizer. For all the time-resolved fluorescence measurements data were collected such that the peak count is >5000.

Experimentally measured time-resolved fluorescence intensity data, F(t), is a convolution of the instrument response function, R(t), and the intensity decay function of the sample, I(t):

1 $$F(t)=\,{\int }_{0}^{t}R(s+\delta )I(t-s)ds$$

where, $\delta$ is the shift parameter, which is a fraction of the time per channel. R(t) is experimentally determined. I(t) is a function assumed to describe the fluorescence dynamics of the sample. Decay data analysis involves the determination of the best values for the unknown parameters in I(t). We fitted the fluorescence decay data from the peak position to the time it decayed to 0.1% of the peak count. Time-resolved fluorescence decay data were fitted to a function that is a sum of discrete exponentials,

2 $$I(t)=\,\sum _{i=1}^{n}{{\alpha }}_{i}\exp (-t/{\tau }_{i})$$

where, *τ*_*i*_ and *α*_*i*_ are the lifetime and the corresponding amplitude, respectively. Also Σ_i_α_i_ = 1.0. There parameters were optimized by the iterative reconvolution method^57^. Correction factors for the parameters (α_i_ and τ_i_.) in successive iterations were determined by the application of Marquardt’s method in nonlinear least-squares analysis^58^. Numerical calculation of the convolution integrals for intensity and partial derivatives were done using the Grinvald-Steinberg recursion equations^57^. The mean lifetime ${\tau }_{m}=\,{\sum }^{}{\alpha }_{i}{\tau }_{i}$, which is the area under I(t) vs t curve, gives us information on the fluorescence quantum yield of the system. Time-resolved measurements of holo- forms of heptamer and monomer azurin were carried out in the presence of 1 mM CuSO_4_ in the buffered solution. Reduction by more than ten times in the *τ*_m_ of holo-azurin heptamer compared to apo-azurin heptamer, in a manner similar to that of the azurin monomer, confirmed copper-binding (Figs 3, S4 and Table S1).

### Single-molecule force spectroscopy (SMFS)

Single-molecule pulling experiments were carried out using a custom-built atomic force microscope, whose details have been described elsewhere^5^. In SMFS pulling experiments, a 50 μl of ~10 μM protein in Tris-HCl (20 mM, pH 7.4) was added on to a glass coverslip. We used gold-coated reflective cantilevers with silicon nitride tip from Bruker, CA, USA. Before acquiring force-versus-extension (FX) traces, spring constant of the cantilever was measured using equipartition theorem as previously suggested by Florin *et al*.^59^. The spring constant of the cantilever that we used was ~40 pN/nm. All the experiments were performed at room temperature and with 5 kHz data sampling rate. All the pulling experiments were performed at a pulling speed of 400 nm/s except for the pulling speed-dependent measurements where it was varied in the range 40–2000 nm/s.

### Data analysis

FX traces were fitted to worm-like chain (WLC) model of polymer elasticity using the Equation 3 given below^60^:

3 $$F(x)=\frac{{k}_{B}T}{p}[\frac{1}{4}{(1-\frac{x}{{L}_{c}})}^{-2}-\frac{1}{4}+\frac{x}{{L}_{c}}]$$

Where, *L*_*c*_ and *p* are contour length and persistence length, respectively. *T* is absolute temperature and *k*_B_ is Boltzmann constant. In the analysis, we considered FX traces that contain complete unravelling of at least four azurin molecules from the heptamer chain.

### Bootstrap method

To estimate the standard error in our experimentally obtained percentage of flux through the intermediate, we carried out the nonparametric bootstrap method that was earlier implemented in analyzing the SMFS data^5,61^. For a given set of experimental FX traces containing a mixture of unfolding pathways (e.g. 2-state and 3-state unfolding for apo-azurin), a sample data set of equal size comprising of randomly chosen FX traces from the experimental set was created. We measured the percentages of traces exhibiting an intermediate for 500 such sample data sets constructed from the original data set. This procedure resulted in a distribution that provided the standard error of the mean for the percentage of traces exhibiting an intermediate and is reported in Fig. 7.

### Azurin models and equilibrium molecular dynamics (MD) simulations

Crystal structures of apo-azurin (PDB code: 1E65) and holo-azurin (PDB code: 4AZU) were processed in Visual Molecular Dynamics (VMD)^62^ to add hydrogen atoms and solvated in a water box of size 153 × 152 × 154 Å^3^. We neutralized the apo-azurin and holo-azurin systems by adding 3 Na^+^ and 2 Na^+^ ions respectively. Subsequently, classical atomistic MD simulations of apo- and holo-azurin protein systems were carried out using the NAMD program (version 2.9)^63^. The simulations employed the TIP3P water model and the CHARMM27 force field to describe atomic interactions^64^. In general, classical force field parameters are not available for metal-ligand coordination spheres and a customized procedure (details in the next subsection) was employed to create multiple models of the copper coordination sphere for holo-azurin. We first equilibrated our protein systems using the following protocol. First, the solvated protein structure was optimized in 20000 steps keeping all the protein heavy atoms (including the copper coordination sphere for holo-azurin) fixed. Then the system was heated from 0 K to 300 K over 50 ps followed by equilibration at 300 K for further 50 ps. The optimization and heating steps were repeated with harmonic constraints on the protein heavy atoms. Subsequently three 150 ps constant pressure and temperature (NPT) equilibration simulations were performed with progressively weaker harmonic constraints. Finally, a single 1 ns run was performed with no constraints. The same equilibration protocol was applied for apo-azurin and the 5 different holo-azurin models described in the next subsection. We then obtained production runs for apo-azurin (100 ns) and for the different holo-azurin systems (10 ns) under constant temperature and volume (NVT) conditions.

### Modelling copper coordination sphere in holo-azurin

At present, there is no force field to mimic the copper coordination sphere in holo-azurin. The trigonal bipyrimidal coordination of copper in holo-azurin with 5 ligands was modelled by a potential comprising of harmonic bonded interactions and non-bonded electrostatic and van der Waals (CHARMM27 parameters) interactions. Atomic partial charges for electrostatic interactions were adapted from van den Bosch *et al*.^65^ (see Table S2). The copper-ligand coordination bonds were modelled as harmonic springs with equilibrium positions set to the crystal structure values (Table S3) and equal spring constants. We varied the spring constant values of the copper-ligand coordination bonds between 1–100 kcal/mol/Å^2^ (1, 5, 25, 100 kcal/mol/Å^2^) to create 4 separate models of holo-azurin. We also created an additional model (no axial) with no constraints on the axial bonds. For the apo-azurin and these 5 holo-azurin systems, we carried out equilibration and production runs as described in the previous subsection.

### Steered molecular dynamics (SMD) simulations

Structures sampled from the equilibrium production runs for apo- and holo- azurin proteins (Fig. S5) were used to seed 10 different SMD simulations in order to build statistics of the unfolding events. In SMD a dummy atom attached by a spring to the terminal protein atom was pulled with a constant velocity of 0.0001 Å/fs. The dummy spring had a spring constant 7 kcal/mol/Å^2^ and extension of this spring was used to monitor the force on the protein. During the pulling, the other terminal protein atom was held fixed so that the pulling direction was along the two terminal atoms of the protein. We carried out both N- and C-terminal pulling simulations wherein azurin was extended. Based on the experimental FX traces and preliminary simulations extending azurin to the full unfolding contour length of ~36 nm (shown in Fig. 2B), we first determined that all experimentally resolved transition states and intermediates would be captured within a molecular extension of 14 nm in the SMD simulations. For a molecular extension of up to 14 nm, we verified that the simulation data was free from artefacts arising from periodic boundary conditions by analysing distances between protein atoms in the simulation cell and the nearest protein atoms of neighbouring protein images. Our analysis shows (Supporting Information Fig. S6) that the distances between protein atoms and their nearest periodic images are always greater than the cut-off distance for non-bonded interactions (1.4 nm) for molecular extensions up to 14 nm.

These N- and C-terminal pulling simulations reveal the structural details of proteins at the atomistic level as they undergo mechanical unfolding. SMD simulations were carried out under NPT conditions using the Langevin dynamics and a Nose-Hoover barostat. The Langevin damping constant was set to 0.01 ps^−1^ after trial SMD runs testing FX traces and temperature controls (Fig. S7). For holo-azurin protein simulations with controlled rupture of copper-ligand bonds, the copper-ligand harmonic bond constraints were removed at different pulling extensions and simulations continued with no constraints to mimic the copper rupture event. Over 100 simulations were carried out on the apo- and holo-azurin proteins (See Table S4 for a listing of systems and simulation timescales).

## Electronic supplementary material

Supplementary Material
