# Supplementary material for: Differences in the mechanical unfolding pathways of apo- and copper-bound azurins
Source: Sci Rep. 2018 Jan 31;8:1989. doi: 10.1038/s41598-018-19755-7 (PMC5792602; doi:10.1038/s41598-018-19755-7)
Supplement: Supplementary file 1 — Supplementary Material [file 41598_2018_19755_MOESM1_ESM.pdf]

Supplementary Material

Differences in the mechanical unfolding pathways of apo- and copper-bound azurins

Anju Yadav, Sanjoy Paul, Ravindra Venkatramani* and Sri Rama Koti Ainavarapu*

Department of Chemical Sciences, Tata Institute of Fundamental Research,

Dr. Homi Bhabha Road, Colaba, Mumbai 400005, India

Corresponding Authors

*Email: ravi.venkatramani@tifr.res.in (R. Venkatramani)

*Email: koti@tifr.res.in (S.R. K. Ainavarapu)

Running Title: Mechanical unfolding of apo- and holo-azurins

KEYWORDS: Mechanical unfolding; Mechanochemistry; Single-molecule force spectroscopy; Steered molecular dynamics simulations, Transition state, Azurin

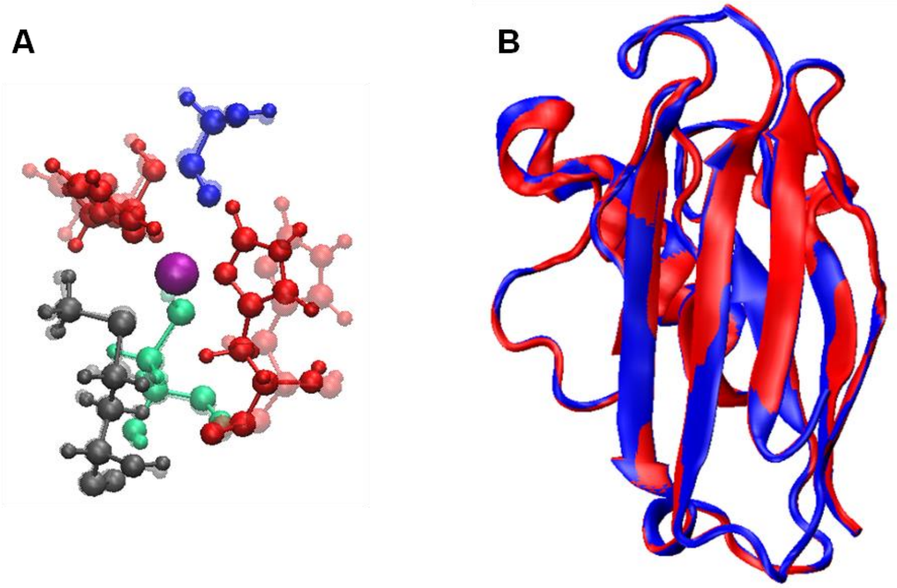

Figure S1 Structural comparison of crystal structures of apo-azurin (PDB code: 1E65) and holo-azurin (PDB code: 4AZU). A, Active site overlap of apo-azurin and holo-azurin. RMSD of active site is 0.6 Å. The copper (purple vdW representation) ligating residues are shown in CPK representation with different colors; Red: Histidine, Blue: Glycine, Green: Cysteine, Grey: Methionine. B, Superposition of apo-azurin (blue) and holo-azurin (red) crystal structures. The RMSD between the structures is 0.2 Å.

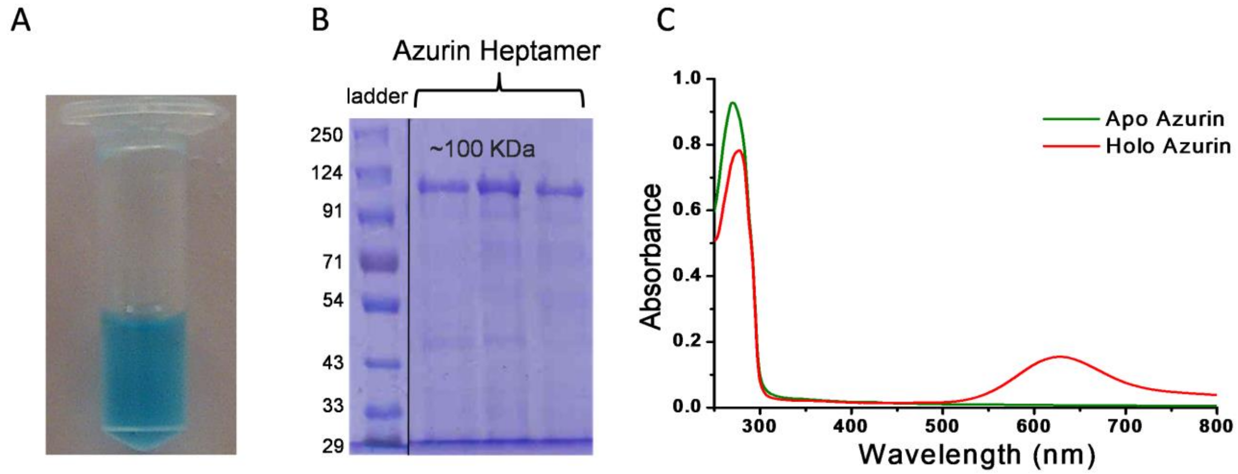

Figure S2 A, Holo-azurin heptamer showing the characteristic blue color. B, SDS-PAGE electrophoresis showing a single-band for azurin-heptamer at ~100 kDa. C, Absorption spectra of azurin heptamer in 20 mM Tris-HCl buffer, pH 7.4. Trp48 of azurin absorbs in the 250-300 nm region. Holo-azurin heptamer has an absorption band centred at 628 nm, which is due to ligand-to-metal charge transfer from S(Cys112) to Cu^{2+} indicating that copper-binding geometry and its coordination sphere properties are unchanged in the azurin-heptamer.

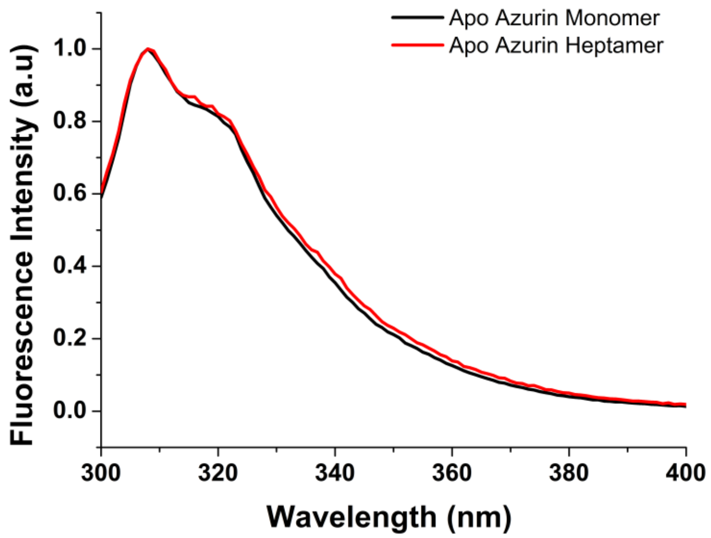

Figure S3 Emission characteristics of Trp48 fluorescence in monomer and heptamer of apo-azurin. The spectra indicate that the structure of azurin is unaltered in the polyprotein compared to its monomer.

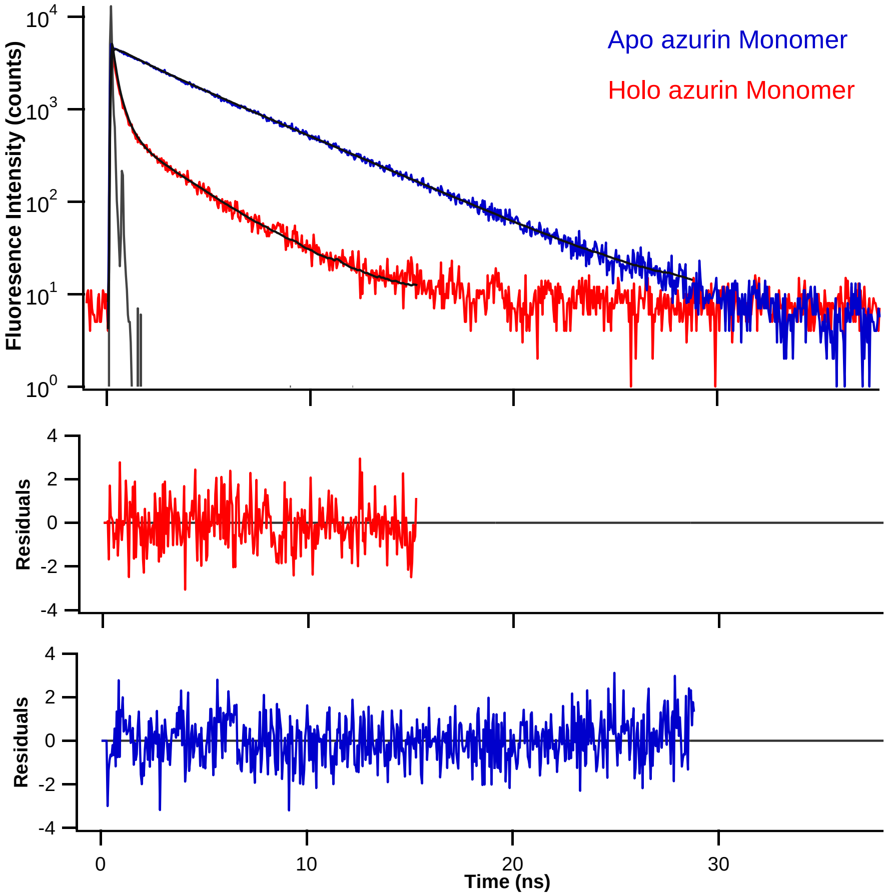

Figure S4. Time-resolved fluorescence intensity of monomers of apo- and holo-azurins. Trp48 in the azurins is excited with 295 nm light. The fluorescence emission was collected at 310 nm. Instrument response function (grey solid-line) and multi-exponential fits to experimental data (black solid-lines) are shown. Residuals are shown in the bottom panels. The parameters of the fits are given in Table S1.

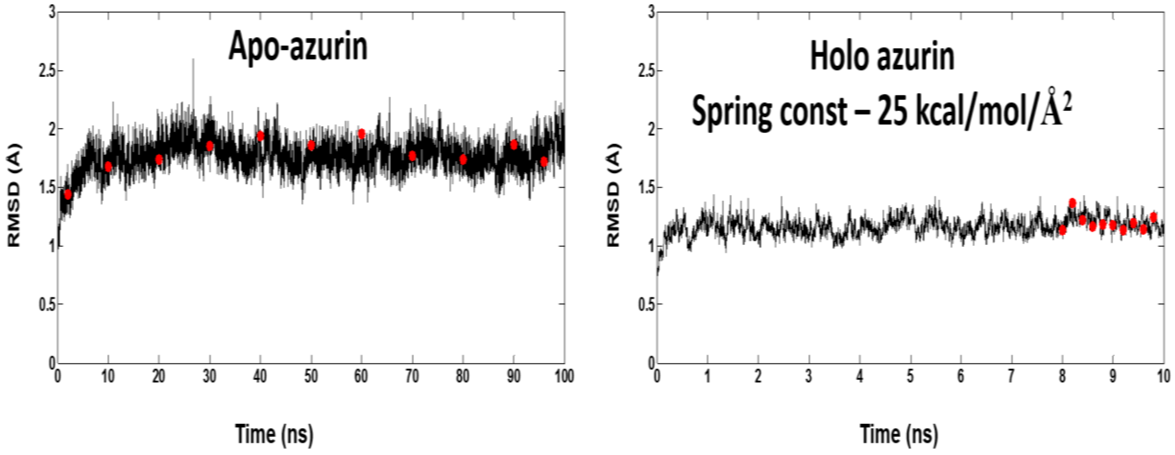

Figure S5 RMSD (backbone) plots for the NVT equilibration trajectories of apo-azurin and holo-azurin. The red markers show the positions of structures seeding the SMD simulations. The holo-azurin plot is for the model with copper coordination bonds constrained with a harmonic spring constant of 25 kcal/mol/Å^2 for which data is discussed in the main manuscript. We followed the same sampling strategy for other holo-azurin models which exhibited similar RMSD plots.

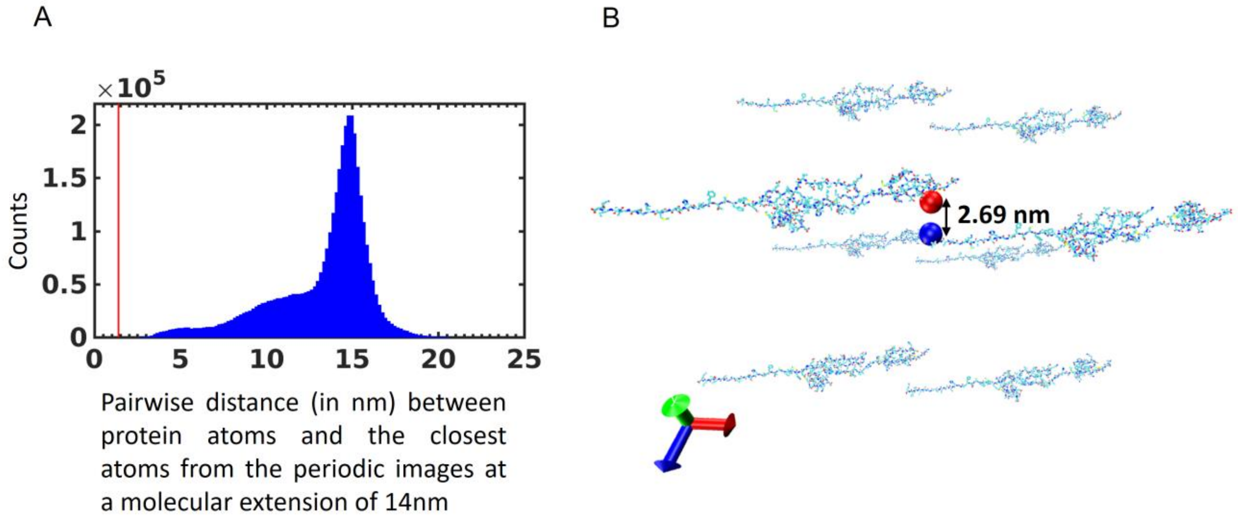

Figure S6 Analysis of our SMD trajectories of azurin show that for molecular extensions up to 14 nm, there is no interaction between protein atoms and their periodic images in our simulation trajectories. A, Distributions of atom-atom pair distances between protein atoms in the simulation unit cell and the closest protein atoms from neighbouring image unit cells. We show

data for an SMD trajectory frame of apo-azurin at 14 nm molecular extension (the largest molecular extension at which simulation data analysis is presented in our study). The vertical red line in the panel indicates the cut-off distance of 1.4 nm for non-bonded interactions employed in our simulations. B, Snapshot of the apo-azurin protein extended by 14 nm in our SMD simulation trajectory. The snapshot shows the protein along with its periodic images and the closest atom-atom separation (2.69 nm) between the protein and an image. Note that the molecular extension is not along the box edges (along the X, Y, Z directions).

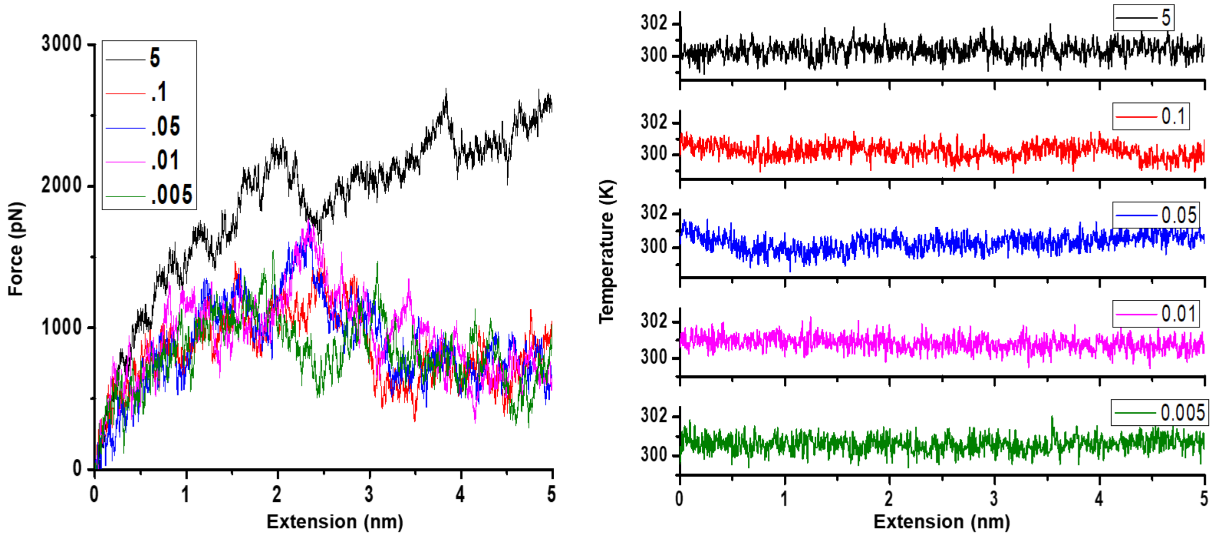

Figure S7 Optimization of Langevin damping (LD) constants. We varied LD values varying from 5 ps^{-1} to 0.005 ps^{-1} (*on left*). The low LD values produce drifts in temperature values, whereas high LD values produce unrealistic FX traces (*on right*). A value of 0.01 ps^{-1} was used for all simulations based on this analysis.

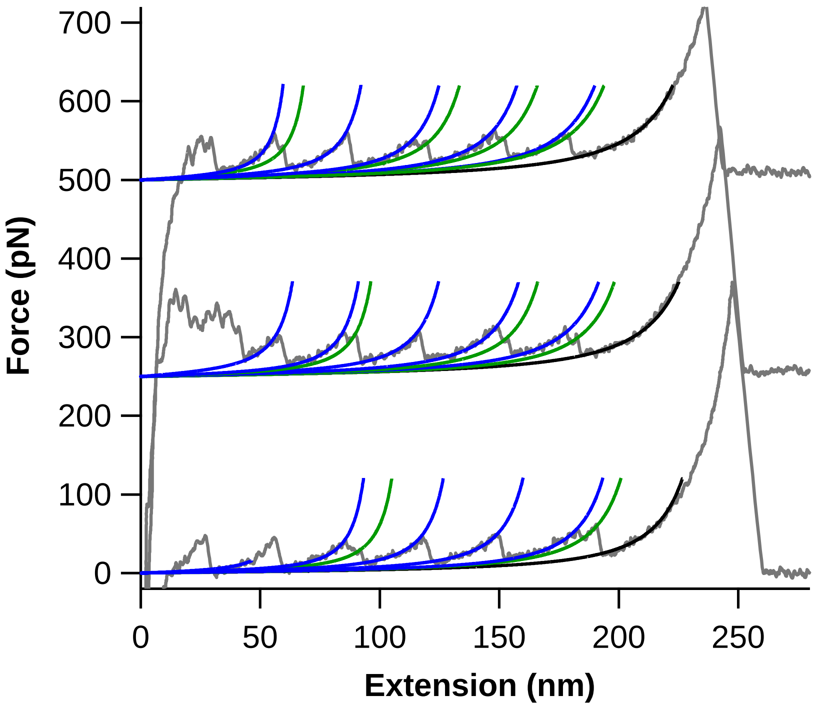

Figure S8 Apo-azurin FX data in addition to that shown in Fig.1 *B*. Top two FX traces are offset along the ordinate by 500 pN and 250 pN, respectively. WLC fits to N_{apo} (blue) and I_{apo} (green) are also shown.

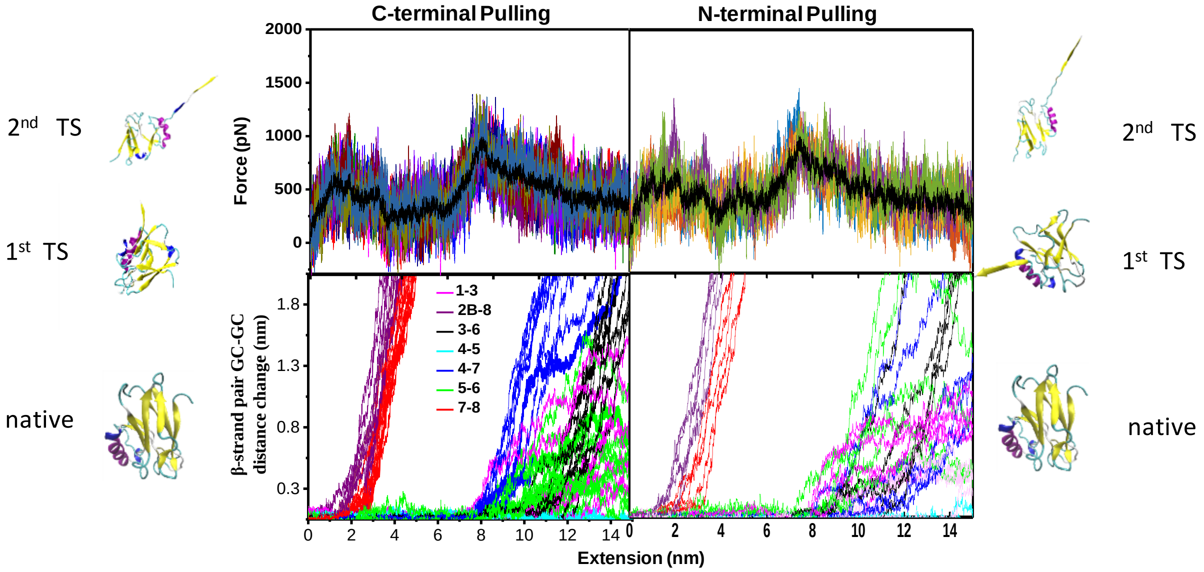

Figure S9 (*Top*) N-terminal and C-terminal SMD pulling of apo-azurin. 10 FX trajectories for C-terminal pulling and 5 FX trajectories for N-terminal pulling mode and their average (solid black line) are shown. Structures of the protein at the beginning, at first TS and second TS are shown. (*Bottom*) Corresponding GC-GC distance change for all the trajectories are shown.

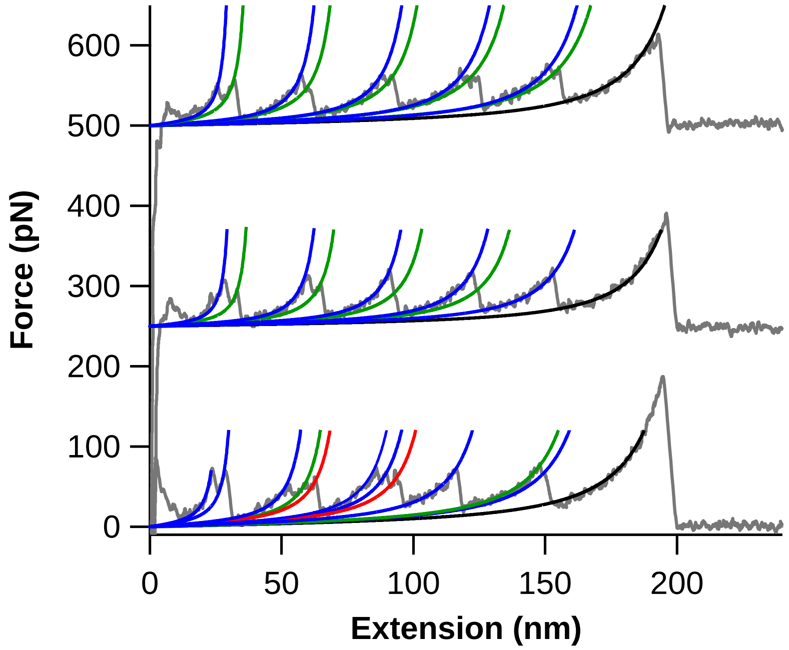

Figure S10 Holo-azurin FX data in addition to that shown in Fig. 4 *A*. Top two FX traces are offset along the ordinate by 500 pN and 250 pN, respectively. WLC fits to N_{holo} (blue), I_{holo} (green) and I'_{holo} (red) are also shown.

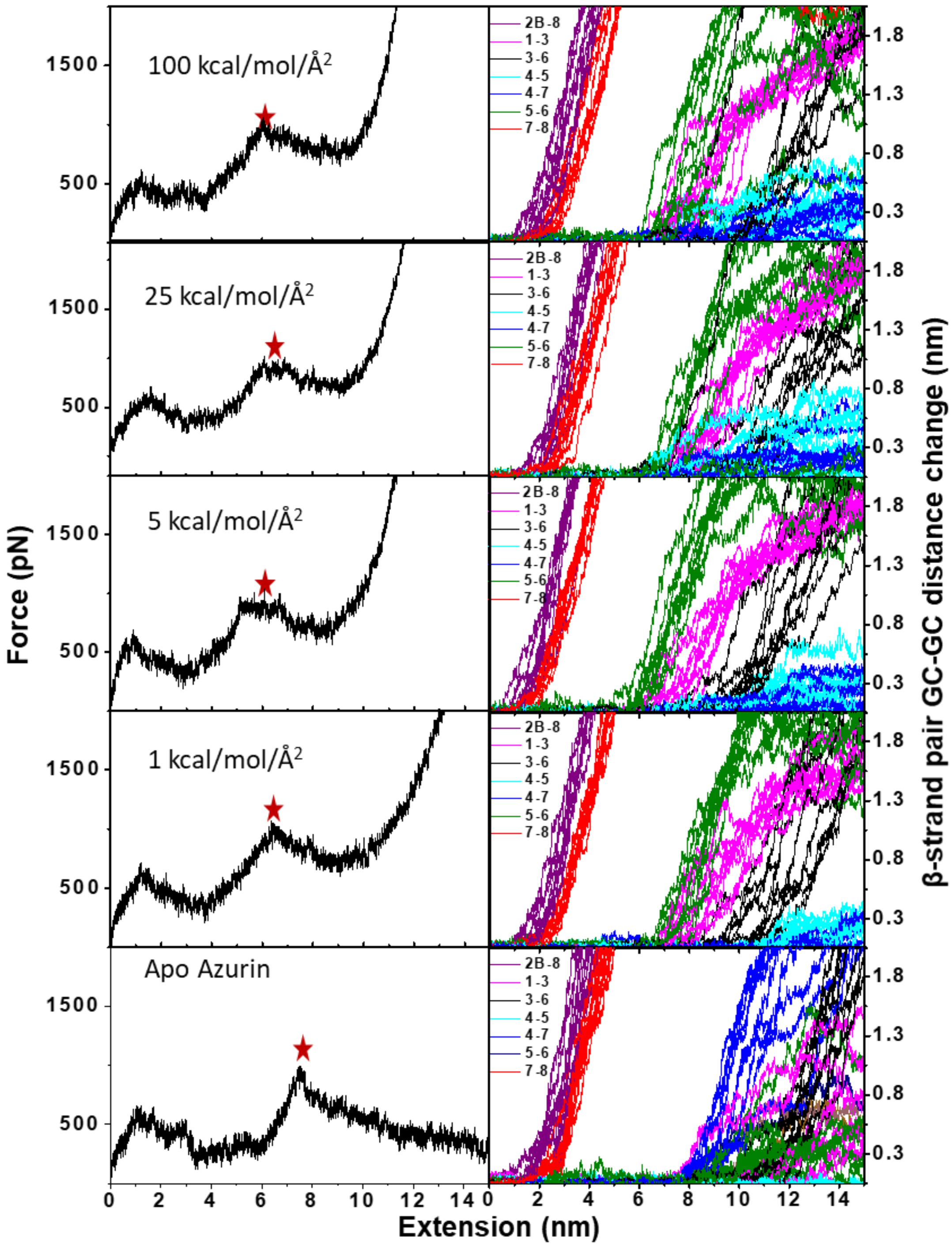

Figure S11 SMD unfolding results for 4 different holo-azurin models with different spring constants (100, 25, 5, 1 kcal/mol/Å^2) for the copper coordination sphere and for apo-azurin. (*Left*) Average SMD FX trace from 10 C-terminal pulling trajectories. (*Right*) GC-GC distance change of β-strand pairs from all 10 trajectories shown simultaneously.

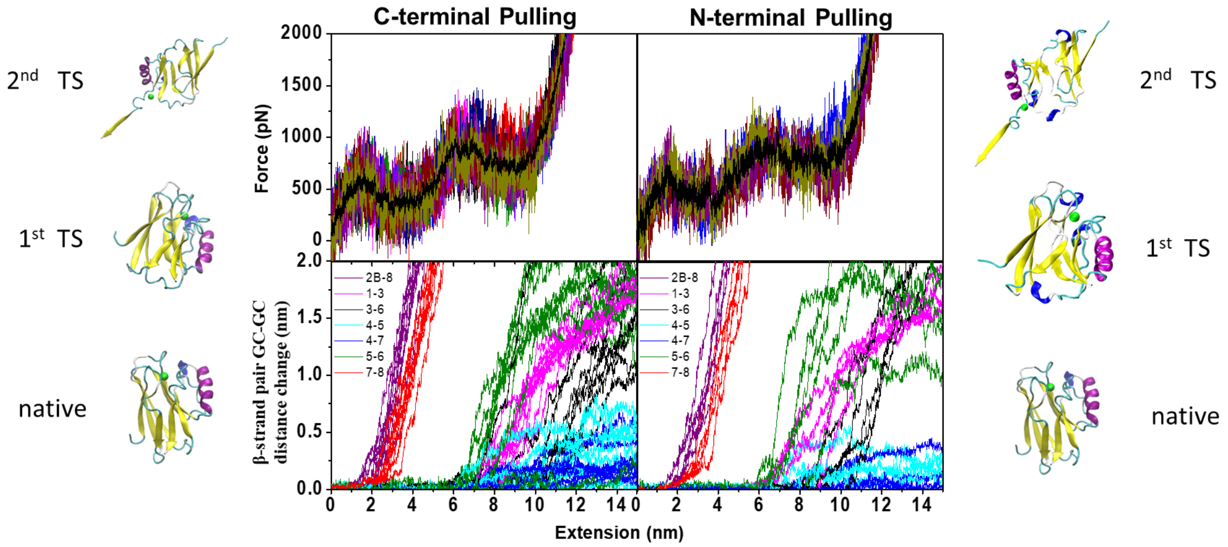

Figure S12 (*Top*) N-terminal and C-terminal SMD pulling simulations of holo-azurin. 10 FX trajectories for C-terminal pulling and 5 FX trajectories for N-terminal pulling mode and their average (solid black line) are shown. Structures of the protein at the beginning, first and second TSs are also shown. (*Bottom*) Corresponding GC-GC elongation for all the trajectories are shown. The spring constant of copper ligand bonds of the coordination sphere are kept at 25 kcal/mol/$Å^2$.

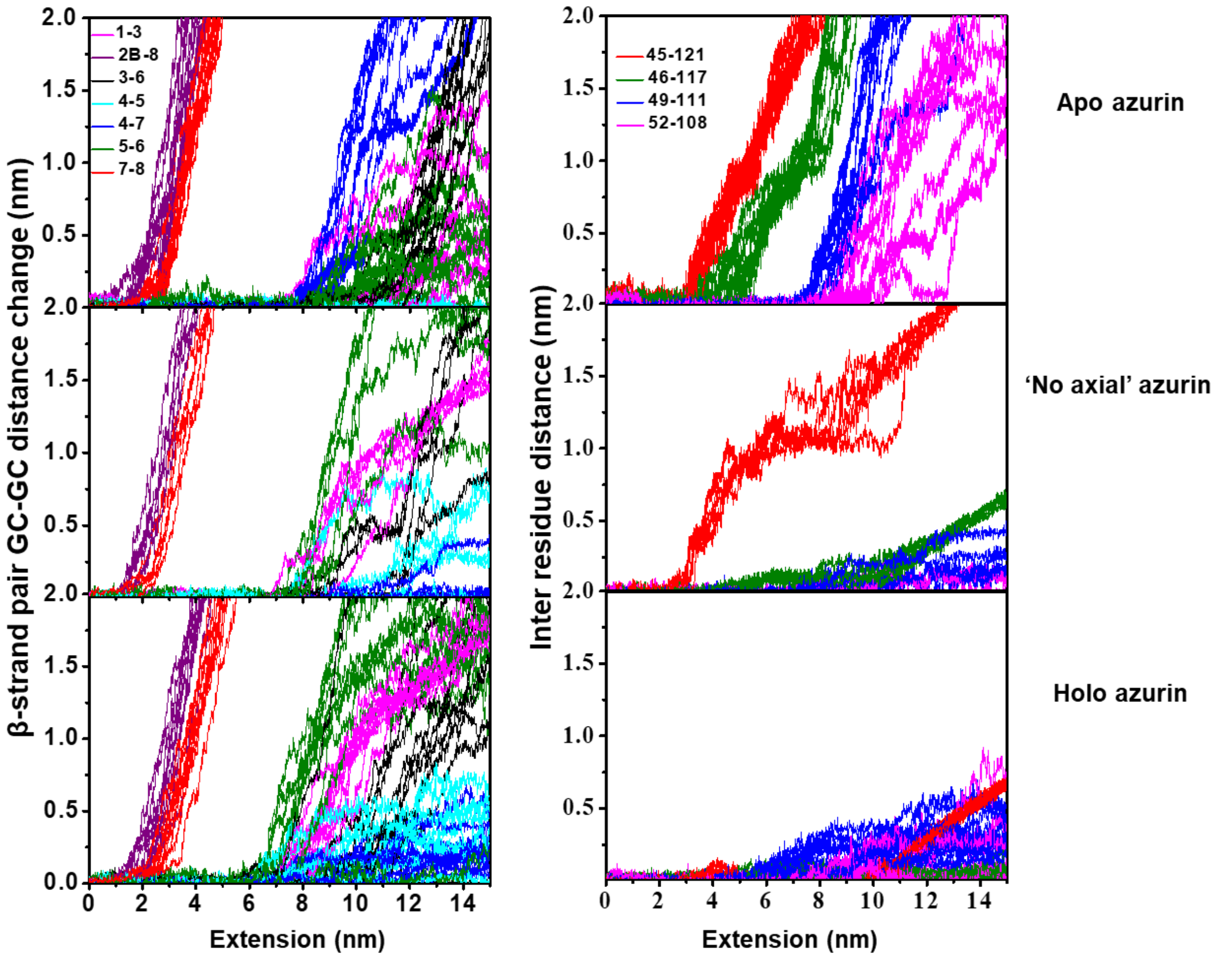

Figure S13 Comparison of GC-GC distance changes and Inter-residue separation for apo-azurin, 'no axial' holo-azurin (25 kcal/mol/Å^2) and holo-azurin (25 kcal/mol/Å^2).

Table S1. Time-resolved fluorescence analysis of different forms of azurin. Non-linear least squares analysis using multi-exponential fits implemented to extract the fluorescence lifetimes (τ) and the corresponding amplitudes (α). Both 2-exp and 3-exp fits were implemented. 2-exp fits were found to be adequate for apo- forms as there is no improvement observed in 3-exp fits (as the χ^2 is not changed significantly). Holo- forms fitted well to 3-exp in comparison to 2-exp.

**Protein**	**τ_1 (ns)**	**τ_2 (ns)**	**τ_3 (ns)**	**α_1**	**α_2**	**α_3**	**τ_m (ns)**	**χ^2**
Apo-azurin heptamer	1.22<br>1.16	4.31<br>3.43	-<br>4.36	0.27<br>0.26	0.73<br>0.08	-<br>0.66	3.48<br>3.45	1.18<br>1.17
Apo-azurin monomer	1.26<br>1.71	4.52<br>4.60	-<br>4.79	0.11<br>0.16	0.89<br>0.59	-<br>0.25	4.16<br>4.19	1.06<br>1.06
Holo-azurin heptamer	0.17<br>0.12	2.38<br>1.01	-<br>3.12	0.91<br>0.89	0.09<br>0.07	-<br>0.04	0.37<br>0.30	2.03<br>1.10
Holo-azurin monomer	0.27<br>0.11	2.74<br>0.39	-<br>2.88	0.91<br>0.61	0.09<br>0.32	-<br>0.07	0.49<br>0.39	1.85<br>1.12

Table S2 Parameters (partial charges and bond distances) used for copper coordination sphere in holo-azurin SMD obtained from Bosch *et al.*[1] The charges provided in Bosch *et al*[1] are for a united atom model. Since we employ an all atom model, we distributed the united atom charges among the heavy atoms and hydrogen atoms while maintaining consistency with the CHARMM force field assignments for specific hydrogen atom types.

**Resid**	**Resname**	**Atom type**	**Partial charge**
45	GLY	N	-0.47
45	GLY	HN	0.31
45	GLY	CA	-0.033
45	GLY	HA1	0.09
45	GLY	HA2	0.09
45	GLY	C	0.4
45	GLY	O	-0.526
46	HSE	N	-0.705
46	HSE	HN	0.384
46	HSE	CA	0.339
46	HSE	HA	0.09
46	HSE	CB	-0.063
46	HSE	HB1	0.09
46	HSE	HB2	0.09
46	HSE	ND1	-0.386
46	HSE	CG	0.079
46	HSE	CE1	0.248
46	HSE	HE1	0.13
46	HSE	NE2	-0.534
46	HSE	HE2	0.494
46	HSE	CD2	-0.035
46	HSE	HD2	0.09
46	HSE	C	0.51
46	HSE	O	-0.51
112	CYS	N	-0.47
112	CYS	HN	0.31
112	CYS	CA	-0.103
112	CYS	HA	0.09
112	CYS	CB	-0.027
112	CYS	HB1	0.09

112	CYS	HB2	0.09
112	CYS	SG	-0.222
112	CYS	C	0.51
112	CYS	O	-0.51
117	HSE	N	-0.47
117	HSE	HN	0.31
117	HSE	CA	-0.107
117	HSE	HA	0.09
117	HSE	CB	0.037
117	HSE	HB1	0.09
117	HSE	HB2	0.09
117	HSE	ND1	-0.391
117	HSE	CG	0.001
117	HSE	CE1	0.236
117	HSE	HE1	0.13
117	HSE	NE2	-0.451
117	HSE	HE2	0.433
117	HSE	CD2	0.091
117	HSE	HD2	0.09
117	HSE	C	0.51
117	HSE	O	-0.51
121	MET	N	-0.47
121	MET	HN	0.31
121	MET	CA	-0.177
121	MET	HA	0.09
121	MET	CB	-0.024
121	MET	HB1	0.09
121	MET	HB2	0.09
121	MET	CG	-0.049
121	MET	HG1	0.09
121	MET	HG2	0.09
121	MET	SD	-0.261
121	MET	CE	-0.129
121	MET	HE1	0.09
121	MET	HE2	0.09
121	MET	HE3	0.09
121	MET	C	0.51
121	MET	O	-0.51
0	CUM	CU	0.971

Table S3 Equilibrium bond lengths of copper-ligand bonds from equilibrium MD runs for the four different holo-azurin models with different harmonic constraints on the copper coordination bonds (columns 2-5). In the last two columns we present crystal structure (PDB code: 4AZU) values of copper-ligand bonds and the equilibrium values used in Bosch *et al*[1] at which we constrained the copper ligand bond lengths.

**Metal-ligand bonds**	**100 kcal/mol/Å^2 (Å)**	**25 kcal/mol/Å^2 (Å)**	**5 kcal/mol/Å^2 (Å)**	**1 kcal/mo/Å^2 (Å)**	**Crystal (Å)**	**Constraining distance from Ref [1] (Å)**
Cu-Gly45:O	2.89 ± 0.05	2.77 ± 0.09	2.62 ± 0.09	2.58 ± 0.09	3.03	2.955
Cu-His46:N	2.44 ± 0.03	2.59 ± 0.05	2.76 ± 0.08	2.87 ± 0.13	2.12	2.064
Cu-Cys112:S	2.64 ± 0.03	2.81 ± 0.05	3.03 ± 0.09	3.20 ± 0.16	2.17	2.267
Cu-His117:N	2.41 ± 0.03	2.57 ± 0.05	2.71 ± 0.07	2.80 ± 0.11	2.00	1.978
Cu-Met121:S	3.18 ± 0.05	3.21 ± 0.09	3.26 ± 0.16	3.38 ± 0.26	3.05	3.164

Table S4 Summary of all systems simulated, number of simulations per system and their timescales.

**System**	**Number of simulations**	**Time per simulation (ns)**	**Total time (ns)**
Apo-azurin equilibrium MD	1	100	100
Holo-azurin equilibrium MD	5	10	50
Apo-azurin SMD (full 40 nm extension)	2	4	8
Apo-azurin SMD (15 nm extension)	10	1.5	15
‘No axial’ azurin SMD	5	1.5	7.5
Apo-azurin N-terminal SMD	5	1.5	7.5
Holo-azurin SMD	40	1.5	60
Holo-azurin copper rupture SMD	20	1.5	30
Holo-azurin N-terminal SMD	5	1.5	7.5
**Total**	**103**		**285.5**

Table S5 2D topology β-strand assignment and sequence of residues used for calculating geometric centre (GC) of β-strands of azurin.

**Strand label**	**Residues**
β1	Resid 4 to 10
β2B	Resid 19 to 22
β3	Resid 28 to 35
β4	Resid 49 to 52
β5	Resid 82 to 83
β6	Resid 91 to 98
β7	Resid 108 to 111
β8	Resid 122 to 127

Table S6 Positions for TS peaks along the molecular extension extracted from average apo- and holo-azurins simulation traces (black solid line) shown in Fig. 2 *C*, Fig. 5 *C*, and Fig. 6. The last two columns show inter-peak separation extracted from this data.

**System**	**1st peak (nm)**	**2nd peak (nm)**	**3rd peak (nm)**	**Inter-peak (1-2) distance (nm)**	**Inter-peak (2-3) distance (nm)**
Apo-azurin	1.3	7.5	-	6.2	-
'No axial' azurin	1.1	6.9		5.8	-
Holo-azurin 100	1.2	6.0		4.8	-
Holo-azurin 25	1.6	6.1		4.5	-
Rupture 4 nm	1.6	7.7		6.1	-
Rupture 6 nm	1.6	5.9	8.6	4.3	2.7
Rupture 7 nm	1.6	5.9	10.6	4.3	4.7
Rupture 8 nm	1.6	5.9	11.7	4.3	5.8

Table S7 Pulling speed dependent intermediate (I_{apo}) flux unfolding through 3-state pathways for apo-azurin.

Speed (nm/sec)	Total no. of events	Events with I_{apo}	Unfolding flux through I_{apo} (%)
40	120	38	32
100	141	74	52
400	315	177	56
1000	133	104	78
2000	124	105	85

Table S8 Pulling speed dependent intermediate (I_{holo}) flux unfolding through 3-state and 4-state pathways for holo-azurin.

Speed (nm/sec)	Total no. of events	Events with I_{holo}	Unfolding flux through I_{holo} (%)
40	100	39	39
100	108	55	51
400	382	295	77
1000	112	97	86
2000	109	77	71

Supplementary Materials and Methods

Azurin monomer expression and purification

The expression of azurin gene in pGK22 vector in *E. coli* BL21 (DE3) cells was induced using 1 mM IPTG after the cell culture optical density at 600 nm (OD_{600}) reached 0.6. The induction was kept at 37^0C for 6 hrs. Azurin monomer (apo-form) was purified using the osmotic shock method accompanied by fractional precipitation steps as described by van de Kamp *et al*[2]. In case of apo-azurin copper sulphate and potassium ferricyanide were not added during fractional precipitation step to the protein containing solution. Both apo- and holo-forms of azurin monomer were further purified using superdex75 columns (GE Healthcare), on Bio-Rad Biologic Duo-Flow FPLC system.

Supplementary References

1. van den Bosch, M. et al. Calculation of the redox potential of the protein azurin and some mutants. *Chembiochem* **6**, 738-46 (2005).
2. van de Kamp, M., Hali, F.C., Rosato, N., Agro, A.F. & Canters, G.W. Purification and characterization of a non-reconstitutable azurin, obtained by heterologous expression of the Pseudomonas aeruginosa azu gene in Escherichia coli. *Biochim Biophys Acta* **1019**, 283-92 (1990).
